# Two‐Dimensional Reconfigurable Photodiode for In‐Sensor Color Filtering and Spectral Logic

**DOI:** 10.1002/adma.72975

**Published:** 2026-04-08

**Authors:** Xiaokun Guo, Yaoqiang Zhou, Yufeng Zhang, Xinyi Zhao, Yue Pang, Lei Tong, Zhipei Sun, Jianbin Xu

**Affiliations:** ^1^ Department of Electronic Engineering and Materials Science and Technology Research Center The Chinese University of Hong Kong Hong Kong SAR China; ^2^ Department of Optical Physics Beijing Institute of Technology Beijing China; ^3^ Department of Electronics and Nanoengineering Aalto University Espoo Finland

**Keywords:** 2D semiconductors, electrically‐tunable filter, in‐sensor processing, organic aggregates, reconfigurable photodiode

## Abstract

Wavelength‐selective photodetectors are essential for applications such as hyperspectral imaging, biomedical diagnostics, and secure optical communication. Conventional photodetection systems typically rely on external filters or post‐processing to resolve spectral information, leading to increased system complexity and data transfer overhead. Here, we report a reconfigurable photodiode based on spatially patterned doped tungsten diselenide (WSe_2_), which exhibits two runtime switchable photodetection modes and a bidirectional wavelength‐dependent conductance modulation across the visible spectrum. Under a broadband photodetection mode, the device exhibited a fast response and a high linear dynamic range of 72 dB. Meanwhile, under color‐filtering mode, the device enables nonvolatile and color‐selective detection spanning from 445 to 780 nm, to experimentally achieve the in‐sensor spectral processing, including color‐based logic operations and object trajectory recognition within the visible wavelength range. We further demonstrate its application in encrypted information identification using chromatically encoded digit patterns, where the device selectively decodes multicolor information via bias‐controlled readout. Simulation results confirm high classification accuracies of approximately 98.99% for red and 98.76% for green patterns using a standard convolutional neural network, highlighting the potential of this platform for hardware‐level spectral‐domain information processing with reduced system complexity.

## Graphical abstract

By harnessing the photodoping of different aggregates, the device exhibits wavelength‐dependent volatile‐to‐nonvolatile photoresponses that can be reconfigured via bias modulation. This enables in‐sensor color filtering and spectral‐encrypted information processing, eliminating reliance on external optical filters or post‐processing algorithms.

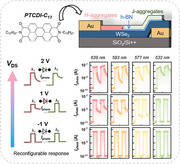

## 1 Introduction

Wavelength‐selective photodetection and imaging are essential for applications such as hyperspectral imaging, biological measurements, autonomous driving, and information encryption. [1, 2, 3, 4] Conventional silicon‐based photodetectors achieve wavelength selectivity through integrating with multiple color‐filtered channels or complex optical structures such as Fabry–Pérot interferometers [5, 6, 7]. However, these solutions often reduce device compactness and limit adaptability to various applications. [8, 9] To address these limitations, two main approaches have been explored from the device point of view: simplifying optical architectures and engineering advanced photoactive materials. On the optical front, introducing electrochromic compounds and spectral reconstruction can reduce the reliance on intricate filtering mechanisms [10, 11, 12], but involves inherent trade‐offs between efficiency and the complexity of heterogeneous integration. In parallel, the development of novel semiconductors—including photosensitive thin film semiconductors [13, 14, 15, 16], colloidal quantum dots [17, 18], nanowire [19], and 2D materials [20, 21, 22] — has opened new possibilities for compact, spectrally selective detectors due to their tunable energy bandgaps and heterogeneous integration capability [23]. By integrating semiconductors of different bandgaps, photodetectors can obviate the need for additional optical filters, enabling selective detection and imaging across various spectral bands, such as near‐infrared to visible [3] or mid‐ to near‐infrared ranges [17]. However, many of these systems rely on discrete junctions and show broad absorption profiles, which limit their ability to achieve continuous wavelength resolution [24, 25].

Additionally, computational reconstruction has been employed to refine wavelength selectivity by leveraging continuous, tunable wavelength‐dependent photoresponses [23]. For instance, tunable band alignment and optoelectronic properties in 2D materials have enabled the development of miniaturized photodetectors with fine spectral resolution across the visible to mid infrared ranges [20, 21, 26]. Yet, most of these platforms encode spectral information implicitly, requiring intensive computation for decoding. This offloads substantial communication burdens onto the peripheral hardware, creating inefficiencies in power and speed [27, 28, 29]. Developing miniaturized wavelength‐selective photodetectors with intelligent data processing and low energy consumption still remains challenging. Inspired by the human retina, integrating signal perception, memory, and processing into a single sensor can mitigate redundant visual data transmission and alleviate computational workload [30, 31]. In particular, 2D material‐based optical sensors, with their tunable energy band alignment and surface‐accessible doping [32, 33], exhibit a wavelength‐dependent bidirectional nonvolatile conductance modulation [34, 35, 36].However, such bidirectional modulation typically requires high‐energy illumination (e.g., ultraviolet light), limiting their capacity for visible‐light color‐sensitive temporal differentiation [37].

In this work, we propose a pattern‐doped WSe_2_ photodiode utilizing dual‐aggregation *N*,*N*′‐ditridecylperylene‐3,4,9,10‐tetracarboxylic diimide (PTCDI‐C_13_) as a photosensitive dopant. The threshold voltage (*V*_TH_) of the PTCDI‐C_13_‐doped WSe_2_ photodiode can be bidirectionally programmed by light with different wavelengths. As a result, the device exhibited two switchable photoresponse modes: When *V*_DS_ ≤ 0 V (photodetection mode), the device operates as a broadband photodiode, exhibiting fast photoresponse over a visible spectral range and a high linear dynamic range of 72 dB. When *V*_DS_ > 0 V (color‐filtering mode), the device can work as an electrically tunable color filter to selectively memory the light with different wavelengths, achieving in‐sensor color processing from 445 to 780 nm. By utilizing positive *V*_DS_ control and delayed readout, we demonstrate that the device achieves reconfigurable color discrimination and color‐based logic operation experimentally without utilizing computational reconstruction. Finally, we validated the device's applicability in encrypted information identification, where chromatically encoded data were selectively decoded through bias‐controlled spectral filtering, achieving simulated classification accuracies of ∼98.99% for red and ∼98.76% for green patterns in a spectrally encoded digit recognition task—enabling hardware‐level secure spectral‐domain information processing.

## 2 Results and Discussion

### 2.1 Bias‐Controlled Photodiode to Photo Memory Transition

**FIGURE 1:**
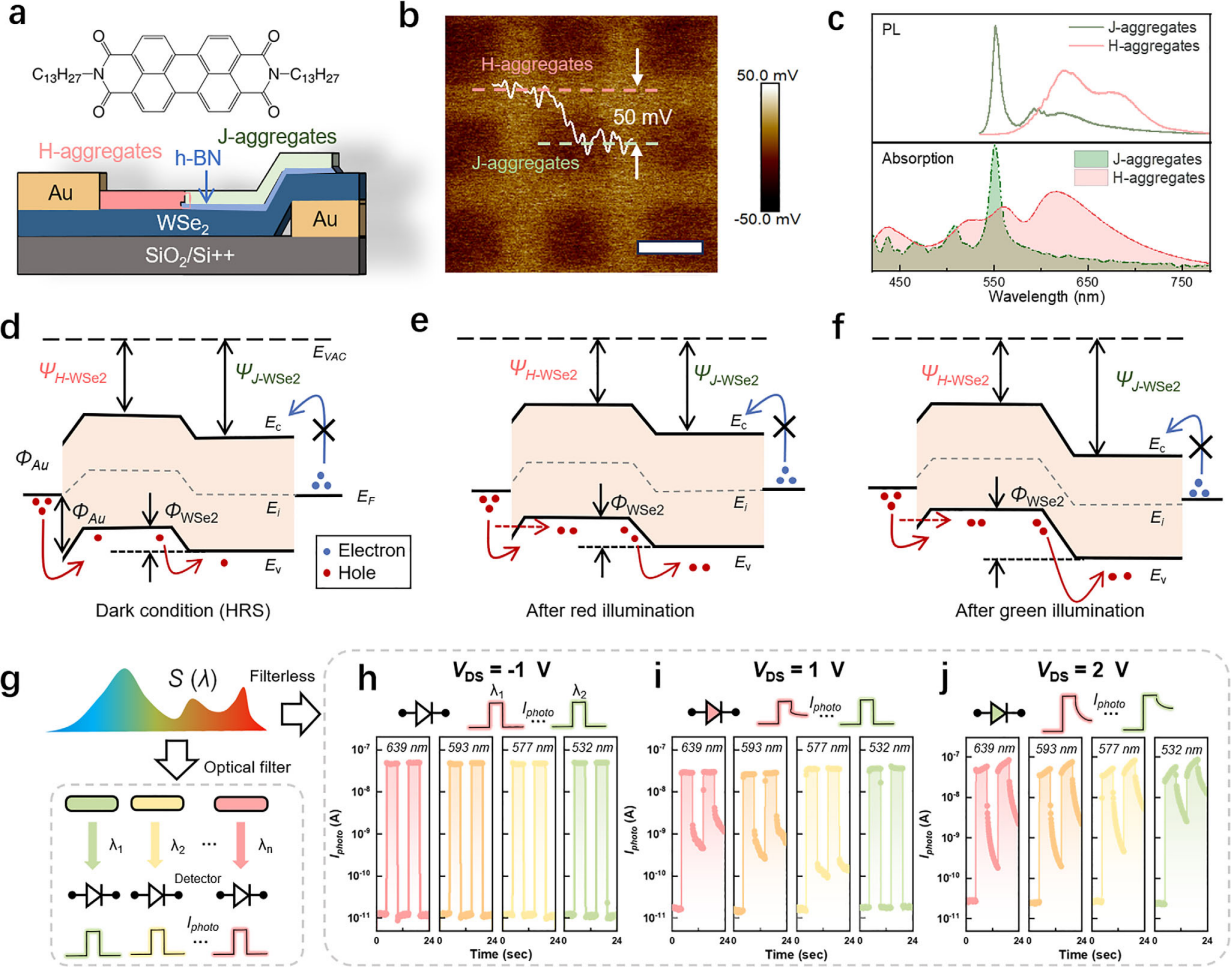
PTCDI‐C_13_‐doped WSe_2_ photodiode concept with *V*_DS_‐controlled volatile to nonvolatile transition. (a) Schematic of the Au offset‐contacted WSe_2_ photodiode with PTCDI‐C_13_ surface doping layer. (b) Surface potential difference measured by Kelvin probe force microscopy, revealing distinct surface doping effect between *J*‐ and *H*‐aggregates. (c) Photoluminescence and absorption spectra of *J*‐aggregate and *H*‐aggregate PTCDI‐C_13_ layers, highlighting the narrow‐band absorption and emission of the *J*‐aggregate in contrast to the broadband absorption of the *H*‐aggregate spanning the entire visible spectrum. (d–f) Schematic energy band diagrams of the device under dark conditions at high resistance state (d), after red illumination (e), and after green illumination (f). The evolution of the band structure under different wavelengths leads to variations in the potential barrier and threshold voltage. (g) Schematics of color‐selective detection based on multiple wavelength filters. Limited by the wavelength transmission range of the filters, multiple wavelength channels are required to achieve color selection within the spectral range. (h–j) Time‐resolved photocurrent during continuous pulse (5 s, 50% duty cycle, 1 mW·cm^−2^) illuminations with varying wavelengths from 639 to 532 nm, biased at *V_DS_* of −1 V (g), 1 V (h), and 2 V (i). These indicate different wavelength‐dependent photo responses as the bias voltage changes.

To realize in situ electrical color filtering via wavelength‐dependent photo memory, we designed and fabricated a spatially patterned doped WSe_2_ phototransistor, in which PTCDI‐C_13_ serves as photosensitive surface dopants to trap the photo‐generated carriers and achieve wavelength‐dependent spectral response, as illustrated in Figure 1a. PTCDI‐C_13_ monomer usually forms two distinct aggregation types—*J*‐aggregates and *H*‐aggregates—depending on its molecular arrangement configuration (Figure S1a). *J*‐aggregates exhibit strong exciton coupling between adjacent molecules [38], resulting in narrow‐band absorption, while *H*‐aggregates, with antiparallel dipole alignments and weaker exciton coupling, display broader absorption profiles due to repulsive excitonic interactions. By utilizing the substrate‐sensitive growth, the *J*‐ and *H*‐aggregate PTCDI‐C_13_ patterns were synthesized via a one‐step physical vapor transport (PVT) method. The detailed growth method is provided in the *Methods* section and further elaborated in Note S1. The Kelvin probe force microscopy (KPFM) measurements reveal a surface potential difference of 50 mV between *J*‐ and *H*‐aggregate PTCDI‐C_13_ (Figure 1b). Compared with the surface potential of WSe_2_ (Figure S2), the *H*‐aggregate exhibits a lower surface potential, serving as the acceptor to achieve the p‐doping to form the WSe_2_ homojunction channel. By leveraging the p‐doping effect of *H*‐aggregate PTCDI‐C_13_, we employed the transferred Au electrode as the *p*‐type electrical contact, showing the flat‐band hole barrier height of ∼200 mV (Figure S3). Additionally, the asymmetric offset Au contact configuration was implemented to ensure single‐sided hole injection from the top contact, enhancing device tunability via the p‐doping effect of the PTCDI‐C_13_ layer. As a result of PTCDI‐C_13_ pattern doping and optimized contact engineering, the device exhibited strong rectifying behavior, with an *I*_DS_‐*V*_DS_ rectification ratio exceeding ∼10^7^ at *V*_GS_ = 0, over five orders of magnitude higher than that of undoped WSe_2_ transistors, as shown in Figure S4. Due to the much lower surface potential of J‐aggregate (Figure 1b), the reduced p‐doping effect leads to an electron current at negative *V_DS_* for the device merely doped with J‐aggregate, which exhibits an asymmetric bidirectional output (green curve in Figure S4). Optical characterizations (Figure 1c) show that *H*‐aggregates exhibit broad absorption and photoluminescence (PL) spectra from 450 to 750 nm due to side‐by‐side dipole alignment, while *J*‐aggregates, with head‐to‐tail dipole orientation, display narrow‐band absorption and PL centered at 550 nm with a full width at half maximum (FWHM) of ∼15 nm (detailed characterization as shown in Note S1).

The distinct absorption characteristics of *J*‐ and *H*‐aggregate PTCDI‐C_13_ lead to different photogating effects and band alignment in WSe_2_, enabling wavelength‐dependent photodoping and optical memory behavior. The device can be modeled as a series connection of a Schottky junction at the Au‐WSe_2_ interface and a homojunction within the channel, as illustrated in Figure S5a, and validated by corresponding surface potential mapping (Figure S5b,c). The schematic energy band diagram in the absence of illumination is shown in Figure 1d. In this initial high resistance state (HRS), the hole transport will be hindered by two main barriers: the contact barrier (*Φ*_Au_) and the homojunction barrier (*Φ*_WSe2_). Under dark conditions, *Φ*_WSe2_ remains relatively small due to the absence of photoinduced doping, *I*_DS_ ‐*V*_DS_ characteristics are mainly determined by *Φ*_Au_, which can be modulated through gate‐voltage pulses. After photoinduced doping, both barriers can be modulated by the charge‐trapping effect of PTCDI‐C_13_. For instance, after red light illumination, e.g., with a wavelength of 639 nm, selective excitation of *H*‐aggregate PTCDI‐C_13_ induces a photogating effect that reduces the contact barrier *Φ*_Au_ by narrowing the band bending width, resulting in a nonvolatile increasing *I*_DS_ when *V*_DS_ is positive, as shown in Figure 1e. In contrast, illumination at 532 nm, which falls within the absorption band of *J*‐aggregates, leads to a PTCDI‐C_13_ downward shift in the surface potential of the WSe_2_, thereby increasing the height of *Φ*_WSe2_ and suppressing charge transport across the homojunction shown in Figure 1f.

Conventional color‐selective detection typically requires multiple optical components, such as optical filters, to select and detect light with targeted wavelengths, as illustrated in Figure 1g. Benefiting from the wavelength‐dependent modulation of band alignment, the pattern‐doped WSe_2_ photodiode enables bias‐controlled nonvolatile photoresponse to a targeted wavelength range without the need for additional optical structures. As illustrated in Figure 1h, under reverse bias conditions (*V*_DS_ ≤ 0 V), the WSe_2_ junction operates as a photodiode, exhibiting a stable broadband volatile photocurrent *I*_photo_. In contrast, when the WSe_2_ junction is forward biased (*V*_DS_ ≥ 0 V), the junction barrier becomes forward biased, and the resistance state becomes highly sensitive to the illumination wavelength, showing excitatory or inhibitory behavior depending on the dominant PTCDI‐C_13_ aggregate (Figure 1i). This behavior stems from the distinct photogating effects of the *J*‐ and *H*‐aggregates, which dynamically reshape the band alignment within the WSe_2_ channel. Although the response amplitude and time constant differ under positive and negative bias conditions, these asymmetric characteristics correspond to distinct operating regimes, with the reverse‐bias regime supporting fast broadband photodetection and the forward‐bias regime enabling wavelength‐dependent nonvolatile switching and color discrimination. Notably, for a fixed illumination wavelength, the nature of the photoresponse—excitatory or inhibitory—can be reconfigured by adjusting *V*_DS_, as shown in Figure 1h–j and Figure S6. This dual tunability in both wavelength and electrical bias allows the device to operate as an electrically reconfigurable color filter, selectively generating photoresponses to specific wavelengths without relying on external optics or filters.

### 2.2 Mechanism of Reconfigurable Photodiode to Photo Memory Transition

**FIGURE 2:**
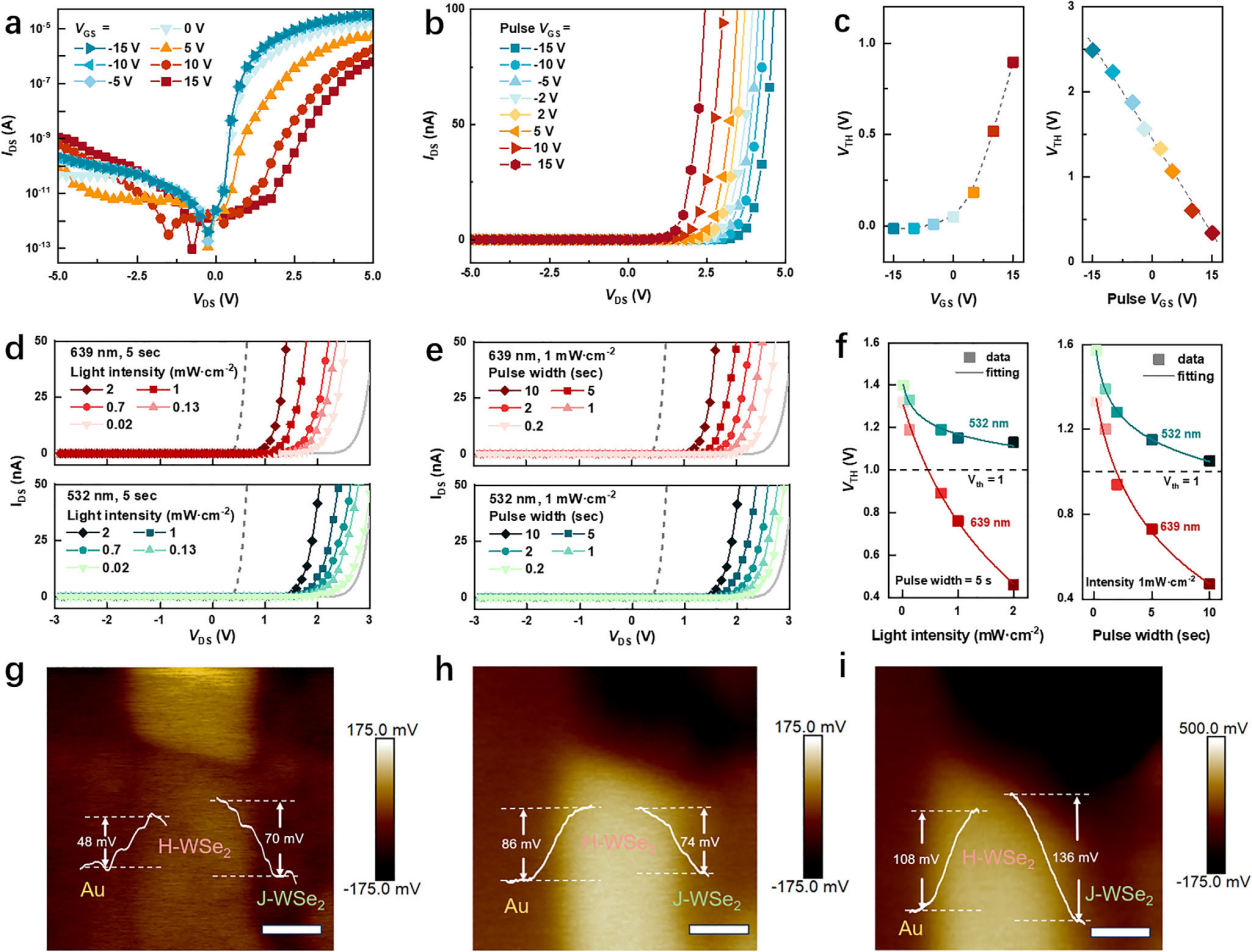
Electrical and optical modulated *V*_TH_ shifting of the WSe_2_ photodiode. (a) Output curves measured at various gate voltages, illustrating electrically tunable *V*_TH_ and rectification ratio of the device. (b) Electrically tunable *V_TH_* shifts observed in the *I_DS_‐V_DS_* curves after applying *V*_GS_ pulses ranging from −15 to 15 V with a duration of 2 s. (c) Extracted *V_TH_* values as a function of applied gate voltages and gate pulses. (d and e) Optically tunable *V_TH_* shifts of the WSe_2_ photodiode observed in the *I_DS_‐V_DS_* curves after laser illuminations of 639 nm (top panel) and 532 nm (bottom panel), at (d) varying light intensities ranging from 0.02 to 2 mW·cm^−2^, and (e) pulse durations from 0.2 to 10 s. (f) Extracted *V_TH_* values exponentially decrease with both light intensity and pulse width, revealing distinct tunable ranges for different illumination wavelengths. (g–i) Surface potential differences of the device measured by KPFM under initial (dashed gray line in Figure 2d) condition (g), after 639 nm illumination (h), and after 532 nm illumination (i). Both with light intensity of 1 mW·cm^−2^ and pulse width of 10 s. Scale bar: 2 µm. These results illustrate significant differences in potential variation of the WSe_2_ homojunction for different wavelength illuminations.

Figure 2a shows the *I*_DS_‐*V*_DS_ curves of the WSe_2_ photodiode, exhibiting a gate‐tunable *V*_TH_ from about 0 to 0.9 V (left panel in Figure 2c) and a rectification ratio ranging from 10^2^ to 10^5^. According to the equivalent circuit model, the *V*_TH_ is primarily determined by the sum of the voltage drop across the contact resistance (*V*_contact_) and the intrinsic threshold voltage of the WSe_2_ homojunction (*V*_TH‐WSe2_), as described in Equation (1).

(1) $$V_{\mathrm{TH}}=V_{\mathrm{TH}-\mathrm{WSe}2}+V_{\mathrm{contact}}\;$$

This relation indicates that *V*_TH_ can be modulated both electrically, via the *V*_GS,_ and optically, through illumination‐induced charge trapping of the PTCDI‐C_13_ layer. To examine the electrical memory effect, we performed dual *V*_GS_ sweeps, revealing unipolar *p*‐type transfer characteristics with the tunable hysteresis loops (Figure S7a). Compared to the undoped device with negligible hysteresis loops (Figure S7b), this memory effect is attributed to the electrical charge trapping effect of the PTCDI‐C_13_ layer. The memory window (Δ*V*) expands with increasing *V*_GS_ sweep range, reaching ∼18.5 V when *V*_GS_ is swept between ± 15 V (Figure S7c). Simultaneously, the resistance switching ratio (HRS/LRS) at *V*_GS_ = 0 V increased exponentially with *V*_DS_, from ∼5 at −2 V to 10^7^ at 2 V, as shown in Figure S7d. The *V*_GS_‐ and *V*_DS_‐tunable memory characteristics enable programmable control of *V*_TH_ for the WSe_2_ photodiode. As shown in Figure 2b, applying programming *V*_GS_ spikes between −15 and 15 V leads to a progressively leftward shift in the *V*_TH_ from 2.51 to 0.35 V (right panel in Figure 2c), attributed to the increased p‐doping effect of the *H*‐aggregate doped WSe_2_ region and a thinner contact barrier width. Beyond electrical programming, optical control of *V*_TH_ is achieved through wavelength‐selective photodoping. Figure 2d‐e shows that 532 nm and 639 nm laser illumination induce power‐ and duration‐dependent shifts in *V*_TH_. For example, under 639 nm illumination, *V*_TH_ decreases from 1.3 to 0.4 V as intensity increases from 0.02 to 2 mW·cm^−2^. In contrast, 532 nm illumination induces a modest shift from 1.4 to 1.13 V. under similar conditions. Likewise, at a fixed power of 1 mW·cm^−2^, increasing the pulse duration from 0.2 to 10 s shifts *V*_TH_ from 1.33 to 0.47 V for 639 nm and from 1.57 to 1.05 V for 532 nm (Figure 2e). A summary of *V*_TH_ shifts as a function of wavelength is shown in Figure 2f, highlighting that 639 nm light produces the most significant modulation. The saturation of *V*_TH_ shift near *V*_DS_ = 1 V under 532 nm illumination provides a basis for its application in color differentiation and encoded signal processing.

To elucidate the mechanism underlying the *V*_TH_ shifting, we performed surface potential mapping after pulsed illumination (10 s, 1 mW·cm^−2^) at 639 and 532 nm, using the initial condition (LRS, indicated by the gray dashed curves in Figure 2d,e) as a baseline (Figure 2g). As shown in Figure 2h, after 639 nm laser pulses, the surface potential difference at the WSe_2_ homojunction interface (Φ_WSe2_) remained nearly unchanged, whereas the surface potential difference at the Au/WSe_2_ interface (Φ_Au_) increased from 48 to 86 mV, indicating a higher contact barrier. Consequently, *V*_TH_ rightward shifted due to the elevated contact resistance. In contrast, 532 nm illumination led to an increase in both ΔΦ_Au/WSe2_ and ΔΦ_WSe2_ (Figure 2i), with ΔΦ_WSe2_ exhibiting a more significant change (from 70 to 136 mV), attributed to the stronger and narrower absorption *J*‐aggregate PTCDI‐C_13_ and its enhanced photogating effect. This increase in ΔΦ_WSe2_ enhances the band bending of WSe_2_, resulting in a more pronounced rightward shifting *V*_TH_ after green light pulses. To further elucidate the wavelength‐dependent photodoping effect, we compared the transconductance of the WSe_2_ phototransistor following 639 and 532 nm laser illumination. The high transconductance (*g*_m_) region reflects carrier transport dominated thermal emission across *Φ*_WSe2_. At *V*_DS_ = 1 V, only 639 nm illumination induced a rightward shift in the *g*_m_‐*V*_GS_ curves (Figure S8c), indicating the Δ*Φ*_WSe2_ was relatively small, in agreement with surface potential measurement. In contrast, at *V*_DS_ = 2 V, both 639 and 532 nm illumination resulted in rightward shifts (Figure S8d), suggesting that under high bias, *Φ*_Au_ rather than *Φ*_WSe2_ became the dominant barrier. Further detailed *g*_m_ analysis is provided in Note S2. The evolution of band alignment under different illumination conditions (dark, 532 nm, 639 nm) and varying *V*_DS_ (−1, 1, 2 V) is further elaborated in Note S3 and Figure S9, showing a decrease in the contact barrier (*Φ*_Au_) upon illumination and an increase in homojunction barrier (*Φ*_WSe2_) at 532 nm illumination or at lower *V*_DS_.

### 2.3 Wavelength‐Dependent Volatile‐to‐Nonvolatile Photo Response Transition

**FIGURE 3:**
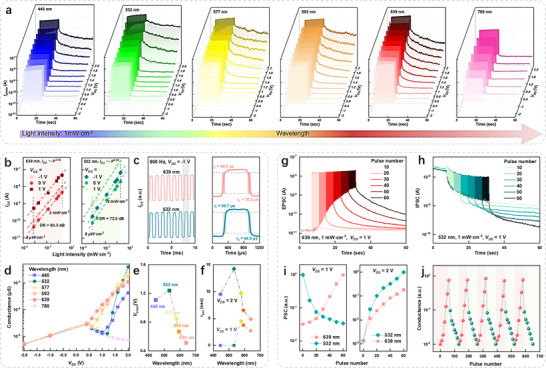
Wavelength‐dependent volatile to nonvolatile photo response transition in the WSe_2_ photodiode. (a) Time‐resolved photocurrent curves at different *V_DS_* after 50 pulses (1 mW·cm^−2^, 5 Hz, 50% duty cycle) of illumination with varying wavelengths ranging from 445 to 780 nm. (b) Photocurrent extracted from the *I_DS_‐V_DS_* curves as a function of light intensity, fitted with a power law. The linear dynamic ranges are 60.3 dB for 639 nm and 72.0 dB for 532 nm, achievable within a light intensity range of 0.008 to 2 mW·cm^−2^ (c) Photo responses and rising/falling times of the device to 639 and 532 nm pulse (800 Hz, 50% duty cycle) signals at *V_DS_* = −1 V. (d) Conductance as a function of *V_DS_* extracted from the postsynaptic current (a) at a fixed decay time of 0.2 s. (e) Transition voltage for the volatile to nonvolatile photo response at different incident wavelengths, corresponding to the transition point of the differential conductance. (f) Decay times fitted from the postsynaptic current at varying incident wavelengths at *V_DS_* of 1 and 2 V. (g) Transition from short‐term potentiation (STP) to long‐term potentiation (LTP) triggered by increasing the number of 639 nm laser pulses (1 mW·cm^−2^, 5 Hz, 50% duty cycle) at *V_DS_* = 1 V. (h) Transition from short‐term depression (STD) to long‐term depression (LTD) triggered by increasing the number of 532 nm laser pulses (1 mW·cm^−2^, 5 Hz, 50% duty cycle) number at *V_DS_* = 1 V. (i) Normalized postsynaptic current after illumination with varying pulse numbers at *V_DS_* of 1 V and 2 V. (j) Cyclic LTP/LTD characteristic curves of the device, triggered by 650 consecutive excitation pulses (639 nm) and inhibition pulses (532 nm) with a frequency of 5 Hz and a 50% duty cycle.

Based on the *V*_TH_ tuning range, the operation mode of the WSe_2_ photodiode can be dynamically switched between a volatile photodetection mode and a wavelength‐dependent nonvolatile photo memory mode by adjusting *V*_DS_. When *V*_DS_ ≤ 0 V, the device served as a traditional photodiode with a broadband and volatile photoresponse, as shown in Figure 3a. Figure S10a–d exhibits light power‐dependent *I*_DS_‐*V*_DS_ curves under 532 and 639 nm illumination. A high *I*_photo_ to dark current *I*_dark_ ratio (*I*_photo_/ *I*_dark_ of ∼10^5^) is observed in reverse bias due to the intrinsic diode behavior. Additionally, the *I*_photo_ increases linearly with incident (Figure 3b), resulting in a high linear dynamic range (*DR*  =  20 · *log*(*I_max_*/*I_min_*)) of 60.3 and 72.0 dB for 639 and 532 nm illumination, respectively. Time‐resolved photoresponse measurements at *V*_DS_ = −1 V (Figure 3c) confirm the stability of the *I*_photo_/ *I*_drak_ ratio under periodic 639 and 532 nm laser pulses at 800 Hz, with fast rise and fall times (τ_r_/τ_d_) of 68.9/76.2 µs for 639 nm illumination and 55.7/64.5 µs for 532 nm. Moreover, due to its photodiode nature, the device demonstrates a self‐powered response, with an open‐circuit voltage (*V*_oc_) ranging from 0.15 to 0.32 V (Figure S10e). To evaluate the photodetection performance under reverse bias, in Figure S10f, we calculate the spectral responsivity *R*_λ_ and detectivity D* at *V*_DS_ = −1 V by using *R*_λ_ = (I_ph_ − *I_D_* ) /*P* · *S*  and D* =  *RA*_0_Δ*f*/*I_n_*, where *P* is the incident optical power, *S* is the effective device area, *A*_0_ is the detector area, *Δ*_f_ is the electrical bandwidth, and *I*_n_ is the noise current. Within the broad 425–775 nm wavelength range, the device exhibits a high *R_λ_* and D* peaking at ∼565 nm due to the strong narrow optical absorption of *J*‐aggregate PTCDI‐C_13_, with the maximum of 14.1 A·W^−1^ and 3 × 10^13^ Jones, comparable to many WSe_2_‐based photodetectors (see Table S1 for a detailed comparison).

When *V*_DS_ > 0 V, the wavelength‐dependent *V*_TH_ shifting induces transitions from volatile to nonvolatile photo response, as shown in Figure 3a,d, enabling an electrical color filtering effect. For example, after 639 nm illuminations (1 mW·cm^−2^, 50 pulses, 5 Hz, 50% duty cycle), the device exhibits a decreasing resistance state when the *V*_DS_ ≥ 0.6 V, defined as the volatile‐to‐nonvolatile transition voltage (*V*_trans_). *V*_trans_ trans exhibits a nonmonotonic dependence on wavelength, reaching a maximum around 532 nm (Figure 3e). In contrast, varying light intensity results in negligible changes in *V*_trans_, exhibiting minimal shifts of only about 0.1 V across the range from 0.02 mW·cm^−2^ to 1 mW·cm^−2^ (Figure S11). This variation is significantly smaller than the *V*_trans_ shift induced by different wavelengths. Additionally, the decay time of WSe_2_ photo memory was also wavelength‐dependent. The decay curve fits a biexponential function: *I_DS_* = *A*_1_ *exp*(− *t*/τ_1_) + *A*_2_ *exp*(− *t*/τ_2_), where, τ_1_ and τ_2_ represent relaxation times associated with the fast and the slow decay processes, respectively. To simplify comparisons, we introduce an average time defined as $\tau_{ave}=\left(A_{1}\tau_{1}^{2}+A_{2}\tau_{2}^{2}\right)\;/\;\left(A_{1}\tau_{1}+A_{2}\tau_{2}\right)$. The results are shown in Figure 3f. The decay rates of the photocurrent after illumination with varying wavelengths differ due to variations in carrier trapping and recombination efficiency. Furthermore, the decay times as a function of incident wavelength can also be modulated by applied bias voltage. At *V_DS_* = 1 V, constrained by the transition threshold, the fitted decay time for 532 nm illumination is extremely lower compared to that for 639 nm illumination (τ_ave_ = 2.75 s). Conversely, as the *V*_DS_ increases to 2 V, the 532 nm illumination produces a longer τ_ave_ of 14.24 s than the 639 nm (τ_ave_ = 4.90 s). By utilizing this modulation of decay time, the photocurrent difference between incident wavelengths can be further optimized.

Notably, unlike conventional photo‐memory devices requiring dual‐band optical stimuli (e.g., ultraviolet and visible light) [39], the reconfigurable photodiode enables fully visible‐spectrum optical control of synaptic behavior. At *V*_DS_ = 1 V, optical stimuli with wavelengths of 639 and 532 nm serve as excitatory and inhibitory presynaptic inputs (EPSI/IPSI), respectively, while *I*_DS_ is monitored as the postsynaptic current (PSC). Upon repeated 639 nm stimulation, PSC is potentiated and retained, mimicking long‐term excitatory plasticity (Figure 3g). Conversely, repeated 532 nm pulses depress PSC, demonstrating inhibitory behavior (Figure 3h). The synaptic weight is further modulated by increasing pulse width or frequency, enabling long‐term potentiation/depression (LTP/LTD) for 639 and 532 nm illumination, respectively, as shown in Figure S12a–d. Additionally, when *V*_DS_ is increased to 2 V (Figure S12e–h), the device enters a unidirectional memory mode: both wavelengths cause EPSC, with retention times remaining wavelength dependent. Figure 3i summarizes the relationship between synaptic modulation and pulse number/width, illustrating symmetric excitatory and inhibitory plasticity. Figure 3j exhibits cycle‐to‐cycle LTP/LTD stability and repeatability at *V*_DS_ = 1 V, where the conductance states were extracted at a fixed delay time of 0.2 s after the termination of optical stimulation. After each readout, the device was reinitialized using a *V*_GS_ pulse, as illustrated in Figure S13. To ensure reliable operation, the reproducibility and reconfigurability of the PTCDI patterned‐doped WSe_2_ photodiode have been validated across multiple devices, exhibiting stable *p*‐type hysteresis, tunable *V*_TH_ modulation, wavelength‐dependent volatile‐to‐nonvolatile transition, and robust cycle‐to‐cycle endurance, as detailed in Note S4 and Figures S14 and S15.

### 2.4 Reconfigurable Color Filtering Effect of the WSe_2_ Photodiode

**FIGURE 4:**
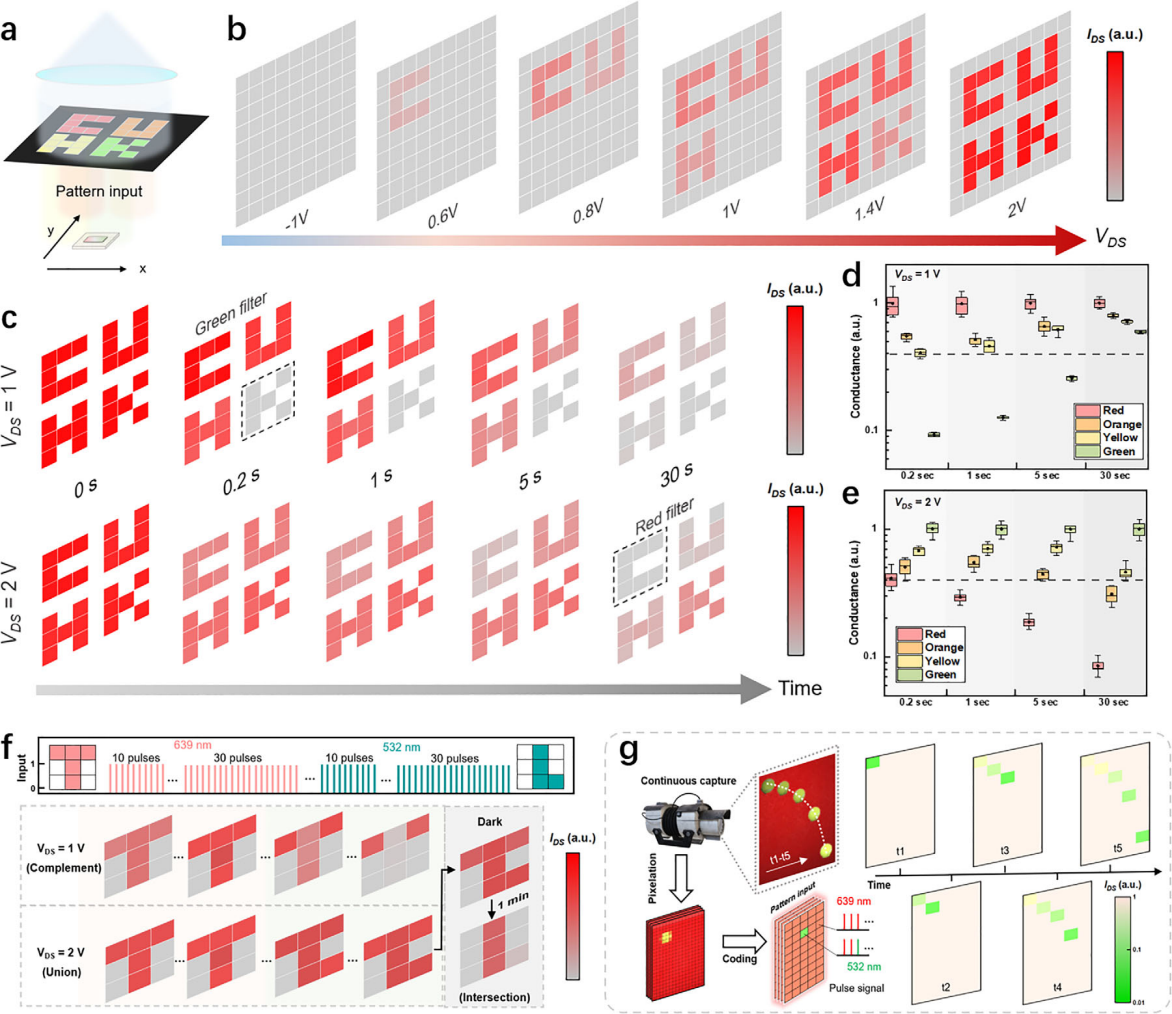
Reconfigurable color filtering effect of the WSe_2_ photodiode. (a) Schematic of the spectral imaging setup. (b) Photocurrent heat maps extracted at a fixed decay time of 0.2 s with varying *V_DS_* from −1 to 2 V, highlighting the device capability in filtering different colors with bias modulation. (c) Photocurrent heat maps extracted at a fixed *V_DS_* of 1 and 2 V with varying decay time ranging from 0 to 30 s. (d and e) Statistics of the device conductance for distinct incident wavelengths at varying decay times when *V*_DS_ = 1 V (d) and 2 V (e). f) Reconfigurable pattern logic operations (intersection, union, complement) implemented by applied laser pulse stimulation. g) Single‐pixel demonstration of color‐selective object motion trajectory recognition based on a green object against a red background.

The wavelength‐dependent volatile‐to‐nonvolatile transition enables the device to function as an electrically tunable color filter, supporting its application in color‐selective optoelectronic systems. To evaluate the color recognition capability of the device, we performed an imaging demonstration by using a single WSe_2_ photodiode with mixed‐wavelength illumination filtered through colored slides (see Methods for imaging details). A patterned metal mask containing semitransparent characters “C,” “U,” “H,” and “K” in red, orange, yellow, and green, respectively, was used to spatially define the incident light, as shown in Figure 4a. The corresponding *I*_DS_ curves were recorded under different *V*_DS_ values (Figure S16), utilizing the wavelength‐dependent photo memory effect to distinguish colors. The colorful pattern is programmed into the continuous optical stimuli, which consists of 50 light pulses, each with a 0.2 s duration and a 50% duty cycle. The delayed reading time *t* is set to 0.2 s, that is, *I*_DS_ is read 0.2 s after the pulse ends. As shown in Figure 4b, at *V*_DS_ = −1 V, the WSe_2_ photodiode exhibits volatile *I*_photo_ with rapid recovery, yielding no persistent response at *t *= 0.2 s. At *V*_DS_ = 0.6 V, the red “C” becomes visible due to nonvolatile photoresponse at the red illumination band. As *V*_DS_ increases to 1.4 V, the orange “U,” yellow “H,” and green “K” patterns emerge sequentially, reflecting the progressive transition voltages of different wavelengths. Consequently, through setting an appropriate *V*_DS_, the specific range of color can be effectively recognized. Beyond electrical tuning, time‐resolved *I*_DS_ measurements also enhance color distinction. Under constant illumination conditions, the device produces a uniform photocurrent regardless of color (Figure 4c). However, after illumination, the *I*_DS_ decay trajectories differ for each wavelength. At *V*_DS_ = 1 V, the device exhibits a relatively fast decay under green light, effectively functioning as a green‐selective filter within a short readout window (0.2–5 s) (Figure 4d). At *V*_DS_ = 2 V, the green‐induced *I*_DS_ persists for a longer duration, producing red‐selective contrast (Figure 4e). Notably, due to the single pixel imaging nature, our device shows ideal cycle‐to‐cycle endurance, reflecting by its narrow interquartile range (Figure 4d,e). By selecting appropriate readout delays, color‐specific responses can thus be extracted from a single exposure.

The dynamic, bidirectional modulation of resistance states also enables reconfigurable logic‐level processing of optical patterns. Devices were initially reset to a high‐resistance state (HRS), and patterned pulses of 639 and 532 nm light were applied in the shapes of “T” and “L,” respectively. At *V*_DS_ = 1 V, the red light acted as a programming input while the green light served to erase the photomemory, resulting in an *I*_DS_ distribution that reflected the logical complement of “T” relative to “L” (Figure 4f). At *V*_DS_ = 2 V, both wavelengths functioned as programming stimuli, yielding a logical union of the two patterns. Moreover, by increasing the delayed reading time, only the overlapping regions of the two inputs remained visible, demonstrating the ability to perform logical intersection. These results establish the WSe_2_ phototransistor as a platform for optical logic operations—complement, union, and intersection—reconfigured by electrical bias. This logic‐level processing can be further used in motion recognition based on color. For example, a green object was thrown in front of a red background, and its trajectory was recorded and encoded into five spatial light patterns (5 × 10 pixels). Then, light patterns were input into the device based on single‐pixel scan imaging. During each frame, only the pixels intersecting with the object's path received 532 nm light, while the background is exposed to constant 639 nm illumination with similar light intensity. This sequence of optical stimuli was applied as five 5‐sec pulses (50% duty cycle). Although the light intensities between target and background are nearly identical, the resulting *I*_DS_ heatmaps captured the parabolic motion of the object (Figure 4g), with color contrast clearly delineating the trajectory. Additionally, the temporal decay profiles of *I*_DS_ reflected the direction of motion, while the current ratios between different spatial locations encoded velocity and acceleration (Figure S17).

### 2.5 Spectrally‐Encrypted Information Processing

**FIGURE 5:**
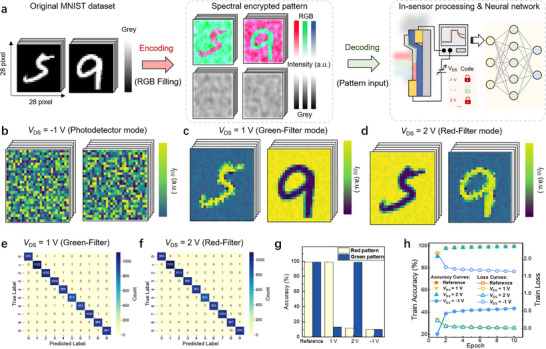
Spectrally‐encrypted information processing. (a) Schematic illustration of the spectral decoding and recognition task, wherein chromatically encoded digit patterns are presented against spectrally contrasting backgrounds. The device is tasked with selectively identifying target digits based on their spectral content. (b–d) Normalized output current *I*_DS_ corresponding to different operational modes of the WSe_2_ photodiode: (b) broadband photodetection mode (*V_DS_* = –1 V), showing no spectral selectivity; (c) red‐selective mode (*V_DS_* = 1 V, readout delay = 0.2 s); (b), green‐selective mode (*V_DS_* = 2 V, readout delay = 1 s). (e,f) Confusion matrices showing digit classification results after 10 training epochs using a CNN trained on grayscale intensity: (e) red‐selective mode; (f) green‐selective mode. (g) Recognition accuracy for red and green digits under different biasing conditions (*V_DS_* = –1 V, 1 V, and 2 V), highlighting the mode‐specific spectral decoding performance. (h) Evolution of classification accuracy during standard and optimized CNN training using *I*_DS_ outputs from the device operated at *V_DS_* = −1 V, 1 V, and 2 V.

Beyond conventional imaging functionalities, the wavelength‐dependent volatile‐to‐nonvolatile transition in the WSe_2_ photodiode presents a framework for in‐sensor spectrally‐encrypted information processing. Compared with conventional photodetector systems that fuse signals from different spectral channels before classification, our device offers a low‐complexity approach to process multicolor signals using a single detector, as illustrated in Figure S18. Although limited long‐term retention and relatively fast decay dynamics may constrain memory [40], they can be exploited for temporal‐domain‐assisted color discrimination, depending on the applied *V*_DS_. The bias‐tunable in‐sensor color filtering enables optical information to be encoded into specific wavelength bands and decoded only under designated electrical readout conditions, allowing direct execution of in‐sensor spectral‐domain processing, which is different from traditional reconfigurable photodiodes that function as programmable synaptic weights in neural networks [41, 42, 43, 44]. To demonstrate this concept, we implemented a wavelength‐selective classification task that mimics multicolor encoded information processing. As illustrated in Figure 5a, the objective is to recognize digit patterns embedded within wavelength‐contrasting backgrounds. To simulate chromatic encoding, we constructed a customized dataset by overlaying red and green channels onto the standard MNIST dataset [45], maintaining equal intensity across images to ensure that spectral, rather than intensity, cues dominate recognition (see Note S5 for details). Digits were encoded in either red or green, forming two distinct chromatic categories. The input signal used in the simulation was derived from experimentally measured device responses under different bias conditions.

In the conventional broadband detection mode (e.g., *V*_DS_ = −1 V), the device exhibits no significant wavelength selectivity due to its broad responsivity and fast relaxation, resulting in indistinguishable photocurrent responses to red and green features. This leads to poor recognition accuracy, as the sensor cannot discriminate between spectrally distinct inputs (Figure 5b). In contrast, by leveraging its *V*_DS_‐dependent photomemory characteristics, the WSe_2_ photodiode can operate as a dynamic color filter. When configured in a “red‐selective” mode (*V*_DS_ = 1 V, readout delay = 0.2 s), the device preferentially responds to red‐encoded features while suppressing green‐channel signals. Under these conditions, red digits can be clearly resolved against green backgrounds, as shown in Figure 5c. Switching to a “green‐selective” mode (*V*_DS_ = 2 V, readout delay = 5 s) inverts the response, allowing selective recognition of green digits embedded in red‐dominated scenes (Figure 5d). Furthermore, by optimizing the *V*_DS_ conditions, a favorable balance between discrimination contrast and readout time can be achieved, yielding up to fivefold higher contrast when increasing *V*_DS_ from 0.6 to 1 V with a fixed readout delay of 0.2 s (Figure S19), potentially boosting throughput without compromising color selectivity. This ability to tune this wavelength weighting on demand eliminates the need for physical color filters or external spectral unmixing processes.

To quantify the effectiveness of in‐sensor spectral decoding, we employed a standard convolutional neural network (CNN) trained solely on grayscale image intensities to classify the chromatically encoded digits [46]. A detailed workflow is provided in Figure S20. The confusion matrices corresponding to red‐ and green‐selective operating modes (Figure 5e,f) reveal classification accuracies of ∼98.99% and ∼98.76%, respectively, comparable to recognition under ideal monochromatic conditions. This improvement stems from the intrinsic wavelength‐selective retention behavior of the device, which enhances contrast between spectral channels while suppressing crosstalk. Class‐wise accuracy analysis (Figure 5g) further confirms mode‐specific decoding behavior: At *V*_DS_ = 1 V, the device accurately identifies red digits but fails on green ones; the response is reversed at *V*_DS_ = 2 V. The spectral discrimination ability is also strongly dependent on the readout delay time, as it modulates the degree of *I*_photo_ retention. Owing to the different decay dynamics of *I*_photo_ under distinct *V*_DS_ conditions, longer integration times either degrade or enhance recognition performance depending on the bias polarity. As shown in Figure S21, at *V*_DS_ = 1 V, increasing the readout delay gradually reduces red‐digit recognition accuracy, dropping to 51.80% after 30 s. Conversely, at *V*_DS_ = 2 V (Figure S22), the accuracy for green‐digit recognition improves with delay time, reaching 99.02% at 30 s. As shown in Figure 5h, even when employing a CNN trained on the *I*_photo_ output pattern from the photodiode at broadband detection mode, the device still yields only ∼40% classification accuracy with high loss after 10 training epochs—significantly lower than the performance of the red‐ and green‐selective modes. These results confirm that the device's reconfigurable, bias‐dependent retention properties enable hardware‐level decoding of chromatically encoded information with minimal computational overhead. With further optimization of device architecture and readout strategies, this approach may be extended toward faster operation for high‐throughput neuromorphic and in‐sensor computing applications.

## 3 Conclusion

In summary, we propose an in‐sensor color discrimination strategy based on a WSe_2_ photodiode with optically tunable *V*_TH_. By leveraging selective substrate‐assisted growth, we achieve spatially resolved doping on the WSe_2_ surface through the self‐assembly of PTCDI‐C_13_ molecules in both *J*‐ and *H*‐aggregate phases, forming a lateral homojunction. This molecularly engineered interface enables two electrically reconfigurable operational modes. In photodetection mode (*V*_DS_ ≤ 0 V), the device exhibits broadband, fast photoresponse with a high dynamic range across the visible spectrum. In the color‐filtering mode (*V*_DS_ > 0 V), the device displays wavelength‐selective, nonvolatile photoresponse, functioning as an electrically tunable color filter operating within the 445–780 nm range. Notably, under fixed positive bias, the resistance state undergoes bidirectional modulation depending on the illumination wavelength. The retention time of these photoinduced states is strongly wavelength‐dependent, which we attribute to the distinct recombination dynamics associated with different PTCDI‐C_13_ molecular aggregates. This bias‐dependent spectral selectivity enables a range of in‐sensor functionalities, including dynamic color recognition, reconfigurable logic operations, and real‐time object trajectory extraction. Additionally, we demonstrate its utility in encrypted information identification. In a spectrally encoded digit recognition task, the photodiode functions as a hardware‐level spectral decoder, selectively extracting chromatically encoded data via voltage‐controlled readout. When integrated with a standard convolutional neural network, the simulation result indicates high classification accuracies—98.99% for red‐encoded and 98.76% for green‐encoded patterns. These results highlight the capability of the device to perform hardware‐level spectral‐domain information processing with low system complexity, offering a promising route toward compact and secure optoelectronic systems.

## 4 Experimental Section

### 4.1 Growth of PTCDI‐C_13_

PTCDI‐C_13_ crystals exhibiting either *J*‐ or *H*‐type aggregation were synthesized via a physical vapor transport (PVT) method. Prior to growth, either mechanically exfoliated h‐BN (HQ Graphene) or CVD‐grown h‐BN films (SixCarbon Technology Shenzhen) were transferred onto SiO_2_/Si substrates to serve as the growth templates. The growth process was conducted in a horizontal tube furnace equipped with a 1‐inch quartz tube (see Figure S1b). PTCDI‐C_13_ powder (Sigma‐Aldrich, purity ≥98%) was placed at the center of the high‐temperature zone, while the prepared substrate was positioned downstream in a lower‐temperature zone. The system was evacuated to a base pressure of ∼2 × 10^−5^ Pa using a molecular pump to minimize contamination and ensure directional vapor transport. The furnace temperature in the source zone was ramped to 230°C and maintained for 90 min. During this period, the substrate temperature was kept below 100°C to facilitate condensation and aggregation. This temperature gradient enabled the formation of *J*‐aggregates on h‐BN regions and *H*‐aggregates on bare SiO_2_. After growth, the system was naturally cooled to room temperature under vacuum.

### 4.2 Device Fabrication

The 2D materials (WSe_2_, h‐BN) were obtained through mechanical exfoliation and stacked using a dry transfer method. Exfoliated few‐layer WSe_2_ and h‐BN were transferred onto a poly(propylene) carbonate (PPC) film supported by a polydimethylsiloxane (PDMS) stamp and then released onto a SiO_2_/Si substrate with an Au bottom contact using acetone. The 2D material arrays were patterned by electron beam lithography (EBL) and reactive ion etching (RIE) (SF_6_, Ar). Then, top electrodes of Au (60 nm) were defined using EBL and deposited by electron beam evaporation on the WSe_2_. Finally, J‐ and H‐aggregate PTCDI‐C_13_ layers were selectively deposited onto the patterned h‐BN/WSe_2_ surface at 230°C for 90 min using the physical vapor transport (PVT) method.

### 4.3 Material Characterization

Raman, absorption, and PL spectra investigation was conducted using the HORIBA LabRAM HR Evolution system with 532 nm laser excitation (the laser spot was ∼1 µm in diameter) and a 100 W while light source. AFM (Bruker, Dimension Icon) in the tapping‐TUNA mode was employed to characteristic the morphonology of the device, while the contact potentials of the different areas were measured via the Kelvin probe force microscopy. Time‐resolved photoluminescence (TRPL) spectra were measured using the HORIBA iHR320 system with 465 nm laser excitation.

### 4.4 Photoelectrical Measurement

The *I*‐*V* characteristics of the device were measured using a Keithley 4200A SCS semiconductor analyzer, equipped with a probe stage and vacuum chamber. Illumination was provided by a set of lasers (445, 532, 577, 593, 639, and 780 nm) and a 300 W Xe lamp with a monochromator (Newport Oriel Cornerstone 260 1/4 m) and an electronic shutter for controlled light exposure.

### 4.5 Signal Pixel Imaging

The device conducts point‐by‐point scanning using motors along the x and y axes. A 8 cm × 8 cm patterned metal mask with a 10 × 10 pixel grid (0.5 cm spacing) featuring semitransparent colored characters “CUHK” was used to define the incident light. To account for the nonvolatile photomemory effect of the device, erasing V_GS_ pulses (−15 V, 2 s) were frequently applied through the bottom gate to reset the resistive state before recording each pixel.

## Conflicts of Interest

The authors declare that they have no competing interests.

## Supporting information




**Supporting File**: adma72975‐sup‐0001‐SuppMat.docx.

## Data Availability

The data that support the findings of this study are available in the supplementary material of this article.

## References

[1] L. Bian , Z. Wang , Y. Zhang , et al., “A Broadband Hyperspectral Image Sensor with High Spatio‐Temporal Resolution,” Nature 635 (2024): 73–81, 10.1038/s41586-024-08109-1. 39506154 PMC11541218

[2] C. P. Bacon , Y. Mattley , and R. DeFrece , “Miniature Spectroscopic Instrumentation: Applications to Biology and Chemistry,” Review of Scientific Instruments 75 (2004): 1–16, 10.1063/1.1633025.

[3] B. Ouyang , J. Wang , G. Zeng , et al., “Bioinspired in‐sensor Spectral Adaptation for Perceiving Spectrally Distinctive Features,” Nature Electronics 7 (2024): 705–713, 10.1038/s41928-024-01208-x.

[4] J. Yoon , “Hyperspectral Imaging for Clinical Applications,” BioChip Journal 16 (2022): 1–12, 10.1007/s13206-021-00041-0.

[5] I. Kim , H. Kim , S. Han , et al., “Metasurfaces‐Driven Hyperspectral Imaging via Multiplexed Plasmonic Resonance Energy Transfer,” Advanced Materials 35 (2023): 2300229, 10.1002/adma.202300229. 37093776

[6] M. Yako , Y. Yamaoka , T. Kiyohara , et al., “Video‐Rate Hyperspectral Camera based on a CMOS‐compatible Random Array of Fabry–Pérot Filters,” Nature Photonics 17 (2023): 218–223, 10.1038/s41566-022-01141-5.

[7] M. J. Grotevent , S. Yakunin , D. Bachmann , et al., “Integrated Photodetectors for Compact Fourier‐Transform Waveguide Spectrometers,” Nature Photonics 17 (2023): 59–64, 10.1038/s41566-022-01088-7. 36628352 PMC9822831

[8] R. A. Crocombe , “Portable Spectroscopy,” Applied spectroscopy 72 (2018): 1701–1751. 30335465 10.1177/0003702818809719

[9] Z. Yang , T. Albrow‐Owen , W. Cai , and T. Hasan , “Miniaturization of Optical Spectrometers,” Science 371 (2021): abe0722. 10.1126/science.abe0722 33509998

[10] J. Bao and M. G. Bawendi , “A Colloidal Quantum Dot Spectrometer,” Nature 523 (2015): 67–70, 10.1038/nature14576. 26135449

[11] S. Zhang , X. Han , X. Liu , et al., “Amorphous‐Crystalline Interface Induced Internal Electric Fields for Electrochromic Smart Window,” Advanced Materials 36 (2024): 2410355, 10.1002/adma.202410355. 39350446

[12] Z. Jia , Y. Sui , L. Qian , et al., “Electrochromic Windows With Fast Response and Wide Dynamic Range for Visible‐Light Modulation Without Traditional Electrodes,” Nature Communications 15 (2024): 6110, 10.1038/s41467-024-50542-3. PMC11271603 39030228

[13] J. Vanderspikken , W. Maes , and K. Vandewal , “Wavelength‐Selective Organic Photodetectors,” Advanced Functional Materials 31 (2021): 2104060, 10.1002/adfm.202104060.

[14] X. He , Y. Li , H. Yu , et al., “A Microsized Optical Spectrometer Based on an Organic Photodetector With an Electrically Tunable Spectral Response,” Nature Electronics 7 (2024): 694–704, 10.1038/s41928-024-01199-9.

[15] Z. Lan , Y. Lei , W. K. E. Chan , S. Chen , D. Luo , and F. Zhu , “Near‐Infrared and Visible Light Dual‐Mode Organic Photodetectors,” Science Advances 6 (2025): aaw8065, 10.1126/sciadv.aaw8065. PMC6994203 32064330

[16] Y. Li , S. Yu , J. Yang , et al., “Filterless Narrowband Photodetectors enabled by Controllable Band Modulation through Ion Migration: The Case of Halide Perovskites,” InfoMat 6 (2024): 12506.

[17] X. Tang , M. M. Ackerman , M. Chen , and P. Guyot‐Sionnest , “Dual‐Band Infrared Imaging Using Stacked Colloidal Quantum Dot Photodiodes,” Nature Photonics 13 (2019): 277–282, 10.1038/s41566-019-0362-1.

[18] J. Kim , C. Jo , M.‐G. Kim , et al., “Vertically Stacked Full Color Quantum Dots Phototransistor Arrays for High‐Resolution and Enhanced Color‐Selective Imaging,” Advanced Materials 34 (2022): 2106215, 10.1002/adma.202106215. 34632653

[19] Z. Yang , H. Cui , F. Gu , et al., “Single‐nanowire Spectrometers,” Science 365 (2019): 1017–1020, 10.1126/science.aax8814. 31488686

[20] S. Yuan , D. Naveh , K. Watanabe , T. Taniguchi , and F. Xia , “A Wavelength‐Scale Black Phosphorus Spectrometer,” Nature Photonics 15 (2021): 601–607, 10.1038/s41566-021-00787-x.

[21] H.‐H. Yoon , H.‐A. Fernandez , F. Nigmatulin , et al., “Miniaturized Spectrometers with a Tunable Van der Waals Junction,” Science 378 (2022): 296–299. 36264793 10.1126/science.add8544

[22] X. Du , Y. Wang , Y. Cui , et al., “A Microspectrometer With Dual‐Signal Spectral Reconstruction,” Nature Electronics 7 (2024): 984–990, 10.1038/s41928-024-01242-9.

[23] S. Yuan , C. Ma , E. Fetaya , et al., “Geometric deep optical sensing,” Science 379 (2025): ade1220. 10.1126/science.ade1220 36927029

[24] P. Wu , L. Ye , L. Tong , et al., “Van der Waals Two‐Color Infrared Photodetector,” Light: Science & Applications 11 (2022): 6, 10.1038/s41377-021-00694-4. PMC8720310 34974520

[25] A. Hwang , M. Park , Y. Park , et al., “Visible and Infrared Dual‐Band Imaging via Ge/MoS_2_ van der Waals Heterostructure,” Science Advances 7 (2025): abj2521, 10.1126/sciadv.abj2521. PMC8673756 34910523

[26] W. Deng , Z. Zheng , J. Li , et al., “Electrically Tunable Two‐Dimensional Heterojunctions for Miniaturized Near‐Infrared Spectrometers,” Nature Communications 13 (2022): 4627, 10.1038/s41467-022-32306-z. PMC9360404 35941126

[27] F. Zhou and Y. Chai , “Near‐Sensor and In‐Sensor Computing,” Nature Electronics 3 (2020): 664–671, 10.1038/s41928-020-00501-9.

[28] Y. Yang , C. Pan , Y. Li , et al., “In‐Sensor Dynamic Computing for Intelligent Machine Vision,” Nature Electronics 7 (2024): 225–233, 10.1038/s41928-024-01124-0.

[29] L. Mennel , J. Symonowicz , S. Wachter , D. K. Polyushkin , A. J. Molina‐Mendoza , and T. Mueller , “Ultrafast Machine Vision With 2D Material Neural Network Image Sensors,” Nature 579 (2020): 62–66, 10.1038/s41586-020-2038-x. 32132692

[30] T. Jiang , Y. Wang , W. Huang , et al., “Retina‐Inspired Organic Neuromorphic Vision Sensor With Polarity Modulation for Decoding Light Information,” Light: Science & Applications 12 (2023): 264, 10.1038/s41377-023-01310-3. PMC10628194 37932276

[31] F. Liao , Z. Zhou , B.‐J. Kim , et al., “Bioinspired in‐sensor Visual Adaptation for Accurate Perception,” Nature Electronics 5 (2022): 84–91, 10.1038/s41928-022-00713-1.

[32] Y. Zhou , L. Tong , Z. Chen , L. Tao , Y. Pang , and J.‐B. Xu , “Contact‐Engineered Reconfigurable Two‐Dimensional Schottky Junction Field‐Effect Transistor With Low Leakage Currents,” Nature Communications 14 (2023): 4270, 10.1038/s41467-023-39705-w. PMC10352327 37460531

[33] Y. Zhou , L. Tong , Z. Chen , et al., “Vertical Nonvolatile Schottky‐Barrier‐Field‐Effect Transistor With Self‐Gating Semimetal Contact,” Advanced Functional Materials 33 (2023): 2213254, 10.1002/adfm.202213254.

[34] Z. Zhang , S. Wang , C. Liu , R. Xie , W. Hu , and P. Zhou , “All‐in‐one Two‐Dimensional Retinomorphic Hardware Device for Motion Detection and Recognition,” Nature Nanotechnology 17 (2022): 27–32, 10.1038/s41565-021-01003-1. 34750561

[35] M.‐Y. Tsai , C.‐T. Huang , C.‐Y. Lin , et al., “A Reconfigurable Transistor and Memory Based on a Two‐Dimensional Heterostructure and Photoinduced Trapping,” Nature Electronics 6 (2023): 755–764, 10.1038/s41928-023-01034-7.

[36] H. Wang , Y. Chen , Z. Ni , and P. Samorì , “An Electrochemical‐Electret Coupled Organic Synapse With Single‐Polarity Driven Reversible Facilitation‐to‐Depression Switching,” Advanced Materials 34 (2022): 2205945, 10.1002/adma.202205945. 36201378

[37] D. Li , Y. Chen , H. Ren , et al., “An Active‐Matrix Synaptic Phototransistor Array for In‐Sensor Spectral Processing,” Advanced Science 11 (2024): 2406401, 10.1002/advs.202406401. 39166499 PMC11497057

[38] X. Zhao , J. Ma , F. Shen , X. Guo , Z. Chen , and J. Xu , “Monolayer J‐Aggregate Crystals Strong Coupling with an All‐Dielectric Metasurface for Photonic Properties Modification,” Laser Photonics Rev 20 (2025): 01208.

[39] D. Cui , M. Pei , Z. Lin , et al., “Versatile Optoelectronic Memristor Based on Wide‐Bandgap Ga_2_O_3_ for Artificial Synapses and Neuromorphic Computing,” Light: Science & Applications 14 (2025): 161, 10.1038/s41377-025-01773-6. PMC11997223 40229240

[40] L. Mennel , J. Symonowicz , S. Wachter , D. K. Polyushkin , A. J. Molina‐Mendoza , and T. Mueller , “Ultrafast Machine Vision With 2D Material Neural Network Image Sensors,” Nature 579 (2020): 62–66, 10.1038/s41586-020-2038-x. 32132692

[41] Z. Zhang , S. Wang , C. Liu , R. Xie , W. Hu , and P. Zhou , “All‐in‐One Two‐Dimensional Retinomorphic Hardware Device for Motion Detection and Recognition,” Nature Nanotechnology 17 (2022): 27–32, 10.1038/s41565-021-01003-1. 34750561

[42] T. Li , J. Miao , X. Fu , et al., “Reconfigurable, nonvolatile Neuromorphic Photovoltaics,” Nature Nanotechnology 18 (2023): 1303–1310, 10.1038/s41565-023-01446-8. 37474683

[43] L. Guo , H. Sun , L. Min , M. Wang , F. Cao , and L. Li , “Two‐Terminal Perovskite Optoelectronic Synapse for Rapid Trained Neuromorphic Computation With High Accuracy,” Advanced Materials 36 (2024): 2402253, 10.1002/adma.202402253. 38553842

[44] G. Wu , X. Zhang , G. Feng , et al., “Ferroelectric‐Defined Reconfigurable Homojunctions for In‐Memory Sensing and Computing,” Nature Materials 22 (2023): 1499–1506, 10.1038/s41563-023-01676-0. 37770677

[45] L. Deng , “The MNIST Database of Handwritten Digit Images for Machine Learning Research [Best of the Web],” IEEE Signal Processing Magazine 29 (2012): 141–142, 10.1109/MSP.2012.2211477.

[46] A. Krizhevsky , I. Sutskever , and G. E. Hinton , “Imagenet Classification with Deep Convolutional Neural Networks,” NeurIPS 25 (2012): 387.

