# Supplementary material for: Two‐Dimensional Reconfigurable Photodiode for In‐Sensor Color Filtering and Spectral Logic
Source: Adv Mater. 2026 Apr 8;38(26):e72975. doi: 10.1002/adma.72975 (PMC13155317; doi:10.1002/adma.72975)
Supplement: Supplementary file 1 — Supporting File: adma72975‐sup‐0001‐SuppMat.docx. [file ADMA-38-e72975-s001.docx]

**Supplementary Information**

**Two-dimensional Reconfigurable Photodiode for In-Sensor Color Filtering and Spectral Logic**

Xiaokun Guo^1^, Yaoqiang Zhou^2,^*, Yufeng Zhang^3^, Xinyi Zhao^1^, Yue Pang^1^, Lei Tong^1^, Zhipei Sun^3,^*, Jianbin Xu^1,^*

1, Department of Electronic Engineering and Materials Science and Technology Research Center, The Chinese University of Hong Kong, Hong Kong SAR, China

2, Department of Optical Physics, Beijing Institute of Technology, China

3, Department of Electronics and Nanoengineering, Aalto University, Finland

***Corresponding author: yaoqiang.zhou@bit.edu.cn; zhipei.sun@aalto.fi; jbxu@ee.cuhk.edu.hk

**Note S1. Aggregation-selected and pattern growth of PTCDI-C_13_**

To realize spatially defined doping in WSe_2_ homojunctions, we developed a substrate-engineered, aggregation-selective growth strategy for PTCDI-C_13_. This organic semiconductor forms two distinct aggregation types—*J*-aggregates and *H*-aggregates—depending on its molecular arrangement configuration (**Fig. S1 a**). *J*-aggregates exhibit strong exciton coupling between adjacent molecules, resulting in narrow-band absorption, while *H*-aggregates, with antiparallel dipole alignments and weaker exciton coupling, display broader absorption profiles due to repulsive excitonic interactions.

Owing to van der Waals heteroepitaxy, PTCDI-C_13_ preferentially assembles into *J*-aggregates on hexagonal boron nitride (h-BN), while *H*-aggregates form on bare SiO_2_. As shown in **Fig. S1 b**， to spatially define these domains, few-layer h-BN was first transferred onto a SiO_2_/Si substrate and patterned via electron beam lithography (EBL), followed by reactive ion etching (RIE) using SF_6_ and Ar gases. The one-step physical vapor transport (PVT) process was then employed to deposit PTCDI-C_13_, yielding selectively patterned regions of *J*- and *H*-aggregates.

Optical microscopy images of h-BN/SiO_2_ before and after PTCDI-C_13_ deposition (**Fig. S1 c-d**) confirm the integrity of the h-BN pattern with well-defined edges. Atomic force microscopy (AFM) (**Fig. S1 e**) indicates an hBN thickness of 2.3 nm. Photoluminescence (PL) mapping identifies distinct emission features from the two aggregate types of PTCDI-C_13_, with peaks centered at ~550 nm for *J*-aggregates and ~630 nm for *H*-aggregates (**Fig. S1 f**), corroborating their spatial and spectral separation.

To further characterize their optical behavior, temperature-dependent PL spectra were collected over a range of 83–293 K. For *J*-aggregates, decreasing temperature suppresses thermal disorder and promotes larger aggregate domains, leading to a marked increase in emission intensity at 551.5 nm (**Fig. S1 g**). *H*-aggregates show similar behavior, with increased intensity and a narrower emission bandwidth upon cooling (**Fig. S1 h**).

The recombination dynamics of photoexcited carriers were further probed via time-resolved photoluminescence (TRPL) spectroscopy (**Fig. S1 i**). *J*-aggregates exhibit a significantly shorter exciton lifetime of 0.53 ns compared to 1.88 ns in *H*-aggregates, indicating more efficient radiative recombination in the former due to enhanced excitonic coupling.

**Note S2. Transconductance analysis**

Here, we measured the transfer curves of the device after exposure to light pulses of 532 nm and 639 nm, both at an identical intensity of 1 mW/cm^2^ and a duration of 5 seconds, as illustrated in **Fig. S7 a-b**. The high resistance state (HRS) and low resistance state (LRS) were programmed using *V*_GS_ pulses of -15 V and 15 V, respectively. At *V*_DS_ = 1 V (**Fig. S7 a**), the transfer threshold voltage (*V*_th_) shifted rightward after exposure to the 532 nm (*V*_th_ = -2.24 V) and 639 nm (*V*_th_ = -1.84 V) lasers compared to the HRS (*V*_th_ = -2.89 V). Notably, the 639 nm illumination induced a larger *V*_th_ shift than the 532 nm light. In contrast, at *V_DS_* = 2 V (**Fig. S7 b**), the *V*_th_ after 532 nm illumination was about -0.69 V, which is higher than that observed with the 639 nm light (*V*_th_ = -1.16 V).

As shown in **Fig. S7 c-d,** for clearer comparison, the transconductance (*g_m_*) curves were calculated using:

$$g_{m}=\frac{\Delta I_{DS}}{\Delta V_{GS}}$$

Generally, a higher *g_m_* value indicates more effective carrier transport across the junction barrier (*Φ*_WSe2_). At a lower *V*_DS_ of 1 V (**Fig. S7 c**), exposure to 639 nm illumination primarily leads to a photodoping effect in the *H*-WSe_2_ region. In this case, hole transport within the valence band traverses a lower *Φ*_WSe2_ in the homojunction, resulting a higher *g_m_* value that approaches the LRS. In contrast, 532 nm illumination photodopes either *H*-WSe_2_ or *J*-WSe_2_ region, leading to an enhanced *Φ*_WSe2_ in the homojunction due to the surface potential difference between *J*- and *H*- aggregates. This higher *Φ*_WSe2_ obstructs the hole transport across the junction region and causes a lower *g_m_* value approaching the HRS. When the *V*_DS_ is increased to 2 V (**Fig. S7 d**), both 639 nm and 532 nm illuminations enhance and rightward shift *g_m_*, approaching the LRS. This is because the larger forward bias partially counteracts *Φ*_WSe2_, facilitating hole transport and increasing *g_m_* under both illumination conditions.

**Note S3. Band alignment evolution**

To explicitly illustrate the wavelength-dependent *V*_TH_ modulation in the WSe_2_ photodiode, we now provide (i) band diagrams at a fixed *V*_DS_ under different illumination wavelengths and (ii) band diagrams at a fixed illumination wavelength under varying *V*_DS_ (**Fig. S9 d-l**). The overall barrier of the device is dominated by a Schottky barrier *Φ*_Au_ at the top-contacted Au/H-WSe_2_ interface and a homojunction within the WSe_2_ channel *Φ*_WSe2_) formed by differential photogating from J- and H-aggregated PTCDI-C_13_. **Fig. S9 a-c** illustrate the photogating effects under dark conditions, 639 nm illumination, and 532 nm illumination, respectively.

Under dark conditions (**Fig. S9d–f**), the device is reset to a high-resistance state (*e.g.*, *V*_TH_ = 2.5 V). Due to varying doping levels induced by different surface potential in the H- and J-aggregate regions, pronounced band bending occurs within the WSe_2_ channel. When a reverse bias (*V_DS_* = −1 V) is applied, the large Schottky barrier prevents carrier injection, resulting in a low *I*_DS_. Even under small forward biases (*V_DS_* = 1-2 V), the strong internal band bending and the reverse-biased Schottky barrier still prevent the device from reaching *V*_TH_, keeping the device in high-resistance state.

After 532 nm illumination, although *Φ*_Au_ is partially reduced, the strong absorption by J-aggregate PTCDI-C_13_ induces significant interface charge transfer, lead to an increase of *Φ*_WSe2_, and a moderate leftward shift in *V*_TH_, as shown by the green curves in **Fig. 2d**. Consequently, at *V_DS_* = −1 V, the junction remains reverse-biased and the device stays in the high-resistance state (**Fig. S9 g and Fig. S6 a**). At *V_DS_* = 1 V, the elevated *Φ*_WSe2_ continues to prevent turn-on (**Fig. S9 h and Fig. S6 b**). However, when *V_DS_* increases to 2 V, the reduced *Φ*_WSe2_ allows the device to reach the threshold condition, leading to increased *I_DS_* and transition to the low-resistance state (**Fig. S9 i and Fig. S6 c**).

After 639 nm illumination, H-aggregate absorption induces significant modulation of *Φ*_Au_, with minimal impact on *Φ*_WSe2_. Although carrier injection is still inhibited when reverse-biased at *V_DS_* = −1 V (**Fig. S9 j**), the lowered Schottky barrier *Φ*_Au_ reduces the effective *V*_TH_, as shown by the red curves in **Fig. 2d**. As a result, under a small forward bias (*e.g.*, *V*_DS_ = 1-2 V), the device can already reach the threshold condition and transition to the low-resistance state (**Fig. S9 j-l and Fig S6 b-c**), giving rise to the observed excitatory behavior.

**Note S4. Reproducibility and reconfigurability evaluation**

To evaluate repeatability and reconfigurability, we fabricated multiple PTCDI-C_13_ patterned doped WSe_2_ photodiodes using the same fabrication process**.** All devices exhibited consistent threshold voltage reconfiguration and reproducible volatile-to-nonvolatile photoresponse transitions, as shown in **Fig. S14 a-d.** The repeatability was systematically evaluated through device-to-device variation analysis:

1. **Electrical characteristics: Fig. S14 e-h** demonstrate the transfer characteristic curves for the devices using varying 𝑉_𝐺𝑆_ sweeping ranges from ±5 V to ±15 V at 𝑉_𝐷𝑆_ = 1 V. All devices exhibit p-type hysteresis with gate-tunable rectification ratios and memory window widths, with values comparable to those of the original device, showing an average rectification ratio of 7.83×10^6^ and a memory window of 18.5 V at V_GS_ sweeping of ±15 V (**Fig. S14 i**).
2. **Tunable threshold voltage modulation:** As shown in **Fig. S14 j-m**, applying programming *V_GS_* spikes between -10 V to 10 V results in a progressively leftward shift in the threshold volage (*V_TH_*) for all devices. The statistics of the extracted 𝑉𝑇𝐻 values indicate a consistent trend, linearly decreasing with the increasing spike amplitude from -10 V to 10 V (**Fig. S14 n**), with an average *V_TH_* value of 2.41 V for the high resistance state (pulsed *V_GS_* = -10 V) and 0.64 V for the low resistance state (pulsed *V_GS_* = 10 V).
3. **Wavelength-dependent volatile-to-nonvolatile transition: Fig. S14 o-r** illustrates photocurrent measurements during continuous pulse illuminations at wavelengths of 639 nm and 532 nm when biased at -1 V, 1 V and 2 V, respectively. For all devices, the transition voltage (*V*_trans_) from volatile to non-volatile photoresponse is observed below 1 V for 639 nm illumination and between 1-2 V for 532 nm illumination. Above the transition threshold for 532 nm illumination (e.g., *V_DS_* = 2 V), a relatively higher photocurrent is obtained compared to that for 639 nm (**Fig. S14 s**). These results are consistent with those measured with the original device, confirming the requirement to use *V_DS_* modulation and delayed readout to discriminate between different colors.

In addition, long-term reconfigurability was evaluated through cycle-to-cycle programming–reading–resetting measurements under 639 nm illumination at *V_DS_* = 1.4 V for 100 cycles. Nearly identical stimulation–decay behaviors were observed across cycles **(Fig. S15 a**). The extracted postsynaptic current (**Fig. S15 b**) remains stable over decay times ranging from 0 to 30 s, demonstrating the endurance and reliable reconfigurability.

**Note S5. Spectrally mixed dataset construction**

To ensure that digit classification relies solely on spectral (color) information rather than intensity variations, we developed an iso-intensity RGB conversion protocol for the MNIST dataset.

First, all grayscale images were normalized to maintain identical total luminance (ΣI = constant). The dataset was then randomly divided into two groups for color encoding: Group A assigned mainly red (R=1.0, G=0, B=0) to pixels with grayscale values >0.6 and mainly green (R=0, G=1.0, B=0) to pixels ≤0.6, while Group B adopted the inverse color scheme. Meanwhile, the blue channel was randomly incorporated as noise signal into the patterns. By strictly controlling the sum of RGB values to 1.0 for each pixel, we guaranteed identical intensity (I=R+G+B) across all pixels, ensuring spectral contrast was determined exclusively by hue (R/G ratio). Validation analysis confirmed all images shared identical intensity distributions (Σ I = 1.0 ± 0.01). This method effectively isolates spectral sensitivity as the sole variable in device testing, prevents intensity-based biases in the CNN classifier, and mimics real-world scenarios where encrypted information is carried by spectral signatures rather than brightness variations.


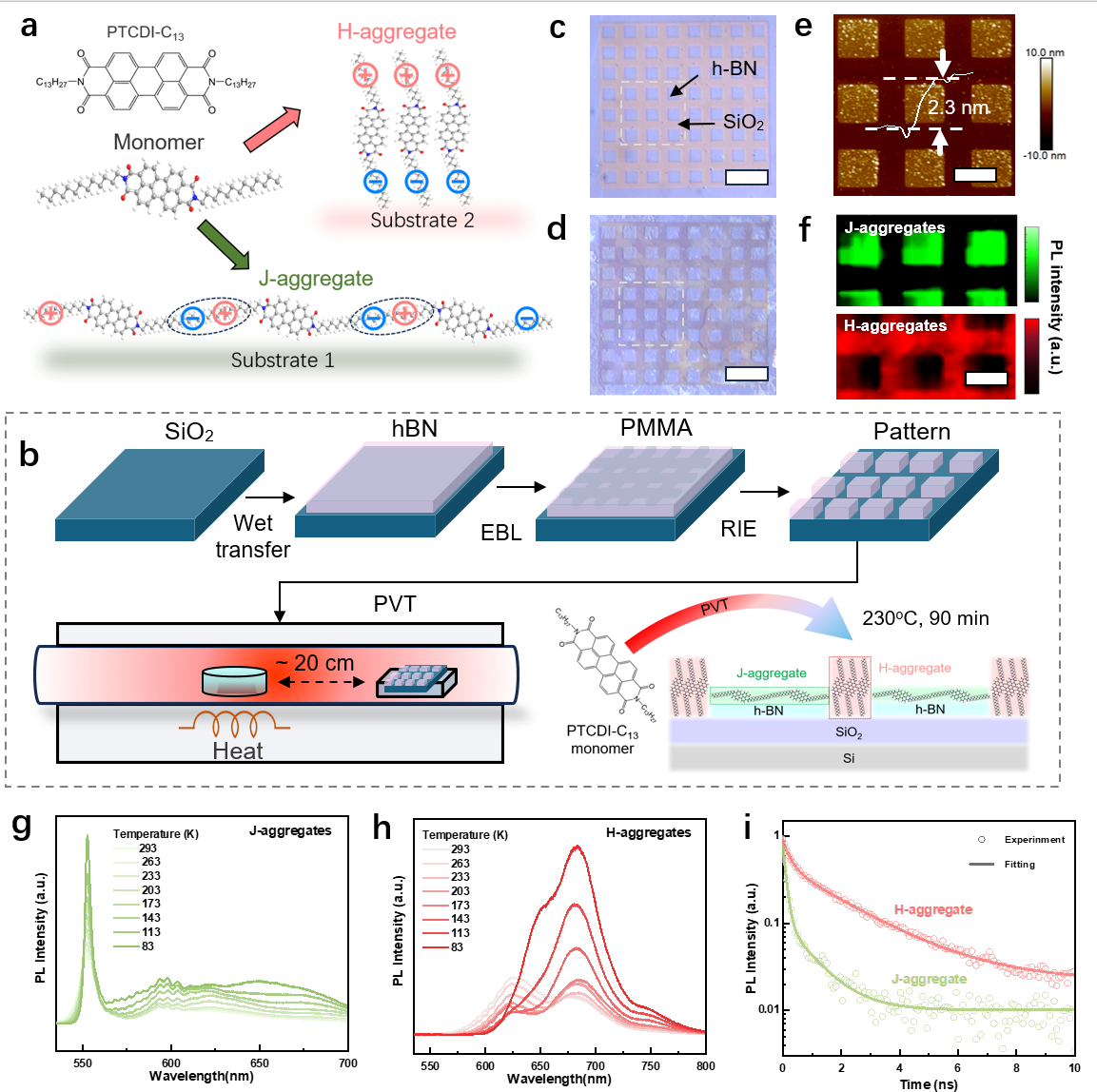


**Fig. S1**. **Aggregation-selected and pattern growth of PTCDI-C_13_**. **a)** Schematic molecular arrangement configuration of PTCDI-C_13_ in monomer, *J*- and *H*- aggregates. **b)** Workflow diagram for aggregation-selected pattern growth of PTCDI-C_13_. **c-d)** Optical images of the patterned h-BN/SiO_2_ substrate before **(c)** and after **(d)** depositing PTCDI-C_13_. Scale bar: 20 μm. **e)** AFM image of the h-BN/SiO_2_ substrate. Scale bar: 5 μm. **f)** PL mapping measured at emission wavelength of 550 nm and 630 nm indicating the distribution of *J*-aggregate and *H*-aggregate, respectively. Scale bar: 5 μm. **g-h)** Low temperature PL spectra of *J*-aggregate **(g)** and *H*-aggregate **(h)** measured with a liquid N_2_ cooling system. **i)** TRPL spectra of the *J*-aggregate and *H*-aggregate. The fitting function of the bi-exponential decay is $R_{t}=A_{1}exp\left( -t/\tau_{1} \right)+A_{2}exp\left( -t/\tau_{2} \right)$. The average lifetime can be calculated using $\tau_{ave}=\left( A_{1}{\tau_{1}}^{2}+A_{2}{\tau_{2}}^{2} \right)/\left( A_{1}\tau_{1}+A_{2}\tau_{2} \right)$. The result shows that the *τ_ave_* of the *J*-aggregate and *H*-aggregate is 0.53 ns and 1.88 ns, respectively.


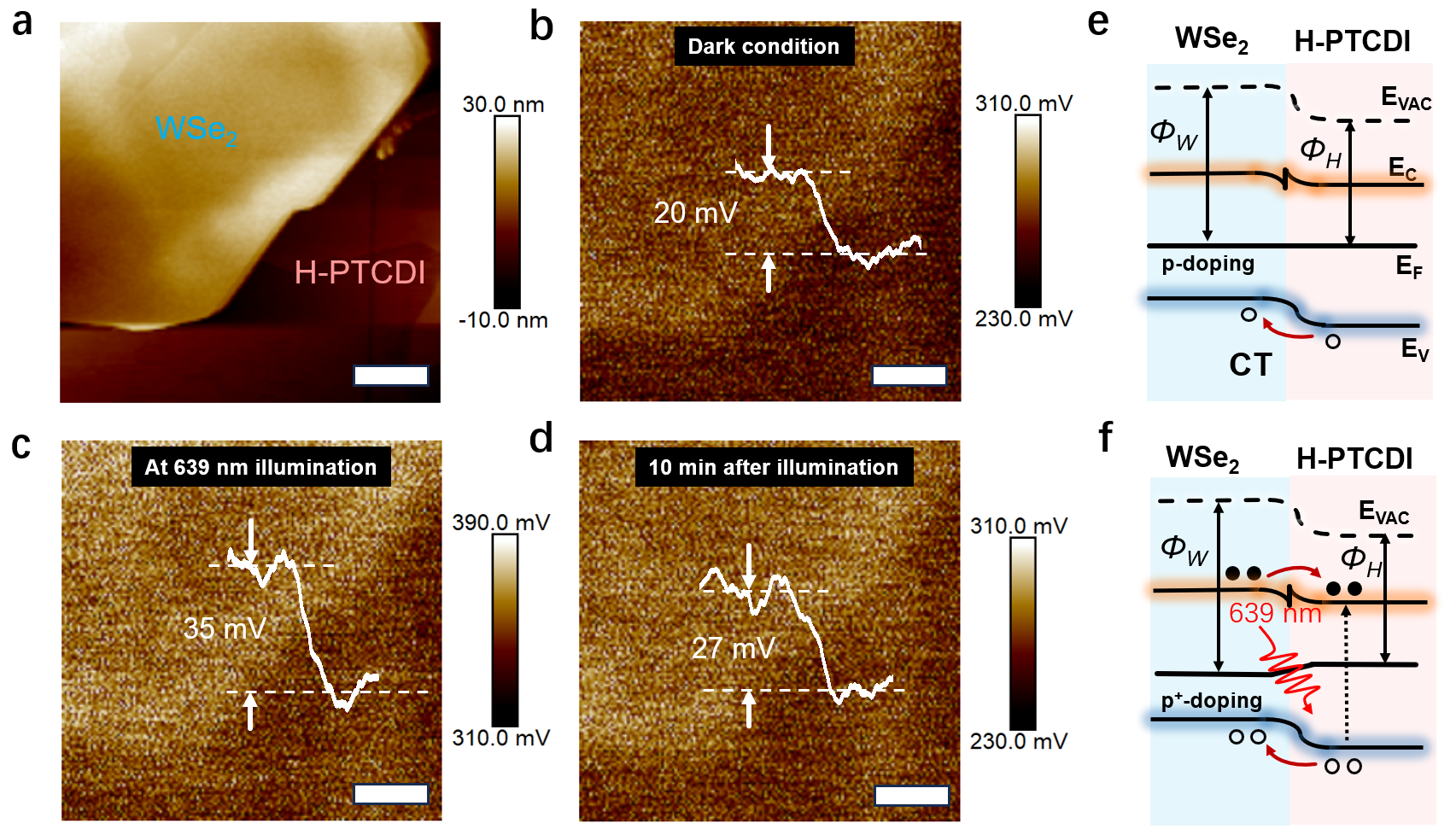


**Fig. S2**. Surface potential difference between WSe_2_ and *H*-PTCDI measured by Kelvin probe force microscopy. **a)** AFM image of WSe_2_/*H*-PTCDI-C_13_ interface. Scale bar: 2 μm. **b-d)** KPFM potential mappings of the WSe_2_/*H*-PTCDI interface under various conditions: dark **(b)**, 639 nm illumination at 1 mW·cm⁻² intensity **(c)**, and 10 minutes after illumination **(d)**. Scale bar: 2 μm. These indicates a p-type surface doping effect of *H-*PTCDI-C_13_ on WSe_2_, which can be enhanced by light illumination and memorized through charge trapping. **e-f)** Schematic band diagrams of the WSe_2_/*H*-PTCDI-C_13_ interface at dark condition **(e)** and at 639 nm illumination **(f)**.


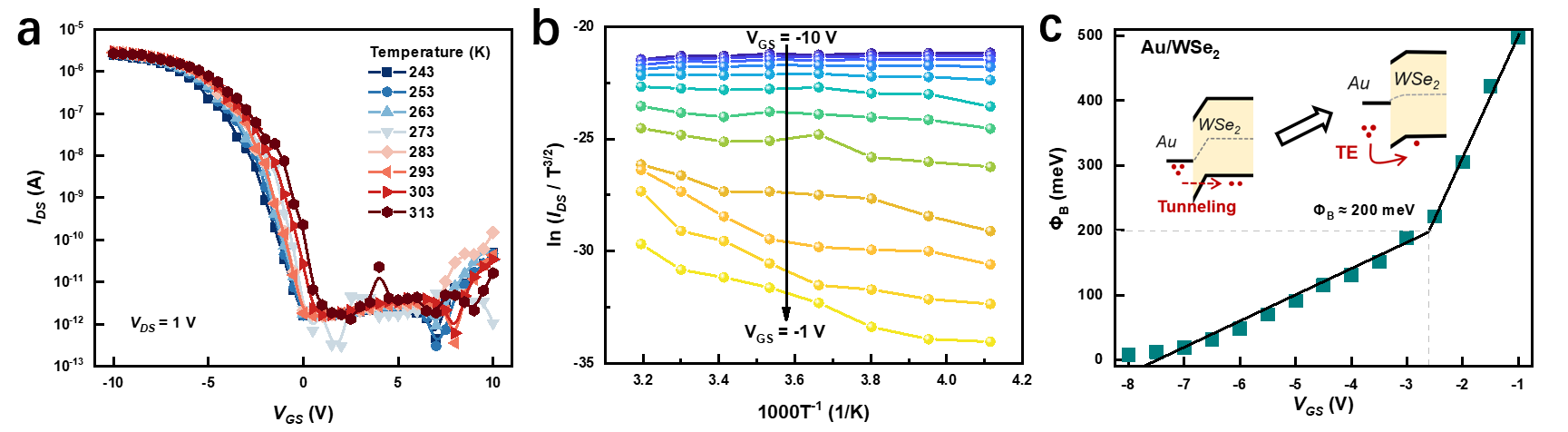


**Fig. S3**. Schottky barrier of Au contacted WSe_2_ is calculated by 2D thermionic emission equation: $I_{DS}=\left[ A^{*}T^{\frac{3}{2}}\exp\left( -\frac{q\Phi_{B}}{k_{B}T} \right) \right]\left[ \exp\left( \frac{qV_{DS}}{k_{B}T}-1 \right) \right]$. Where *I_DS_* is the saturation current density, *A** is the effective Richardson–Boltzmann constant, *T* is temperature, *q* is the electron charge, *k_B_* is the Boltzmann constant, and *Φ_B_* is the Schottky barrier height. The Schottky barrier height is extracted under a flat-band gate voltage condition, which was responsible for the start of deviations from the linear behavior. **a)** *I*_DS_-*V*_GS_ curves of Au-WSe_2_ transistor biased at 1 V with varying temperatures ranging from 313 K to 243 K. **b)** Arrhenius plots of *ln (I_DS_ /T^3/2^)* versus *1000/T* at varying gate voltages from -10 V to -1 V of Au-WSe_2_ contact. **c)** Barrier heights of the Au-WSe_2_ Schottky junctions as a function of *V*_GS_. The Schottky barrier height is extracted under a flat-band voltage.


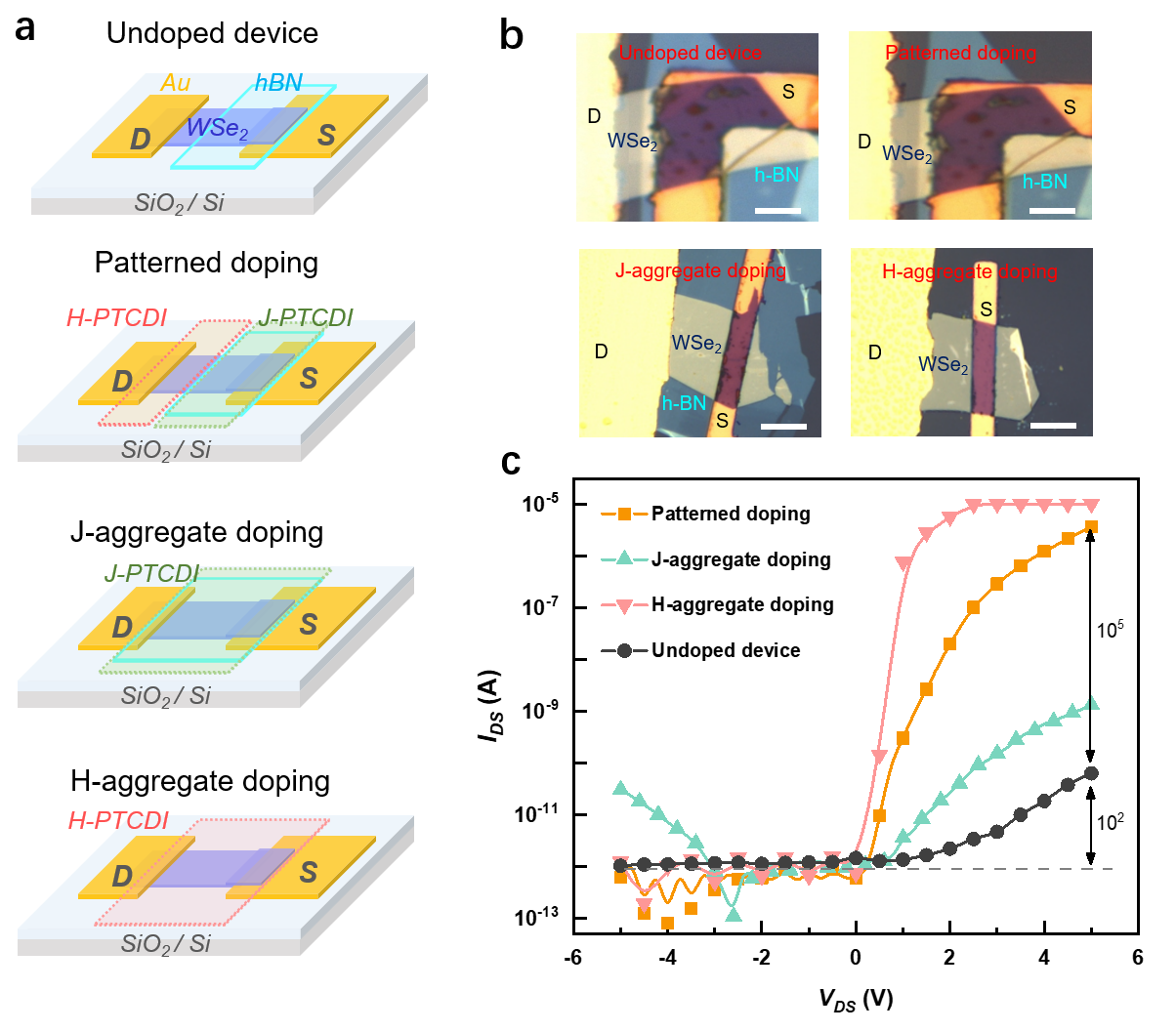


**Fig. S4**. **a-b)** Schematics **(a)** and optical images **(b)** or the Au offset-contacted WSe_2_ diode under different surface doping conditions (undoped, patterned doping, J-aggregate doping and H-aggregate doping, as described in each panel, scale bar: 5 μm). **c)** *I_DS_-V_DS_* curves of the Au offset-contacted WSe_2_ diodes before (black curve) and after (colored curves) depositing PTCDI-C_13_ layers. Large rectification ratios of over 10^7^ is obtained after doping with either patterned PTCDI or H-aggregate PTCDI, significantly higher than the undoped device (~10^2^).


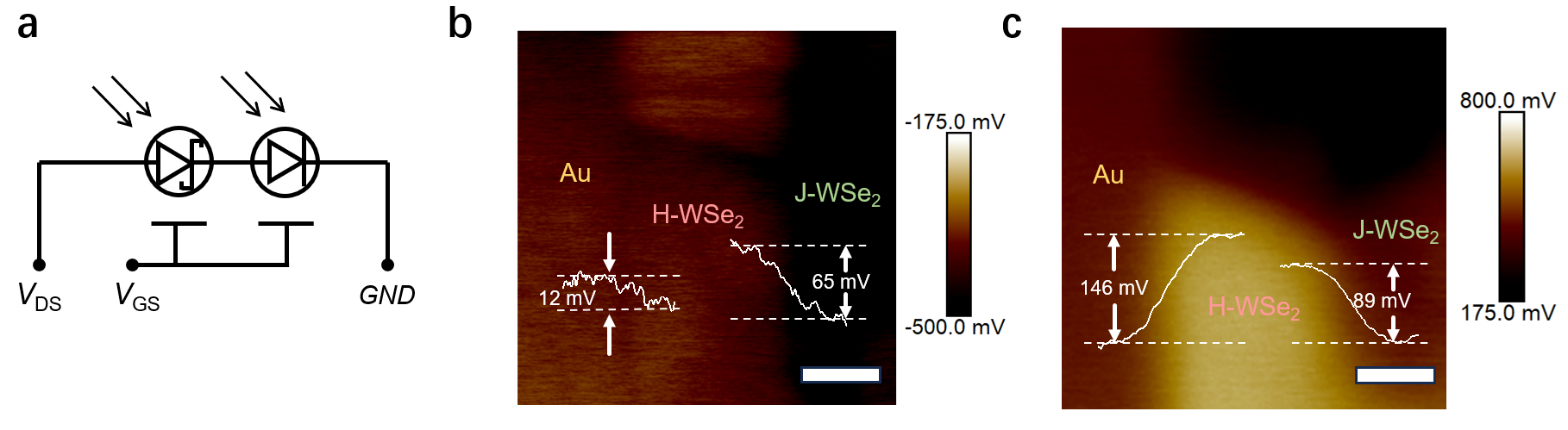


**Fig. S5**. **a)** Schematic circuit diagram of the device as a series connection of a Schottky junction at the Au-WSe_2_ interface and a homojunction within the doped WSe_2_ channel. **b-c)** Surface potential differences measured by Kelvin probe force microscopy after applying *V*_GS_ pulses of 15 V **(b)** and -15 V **(c)**. Scale bar: 2 μm. These measurements reveal the existence and gate tunability of both the Schottky barrier and the homojunction barrier, corresponding to the dual-junction configuration.


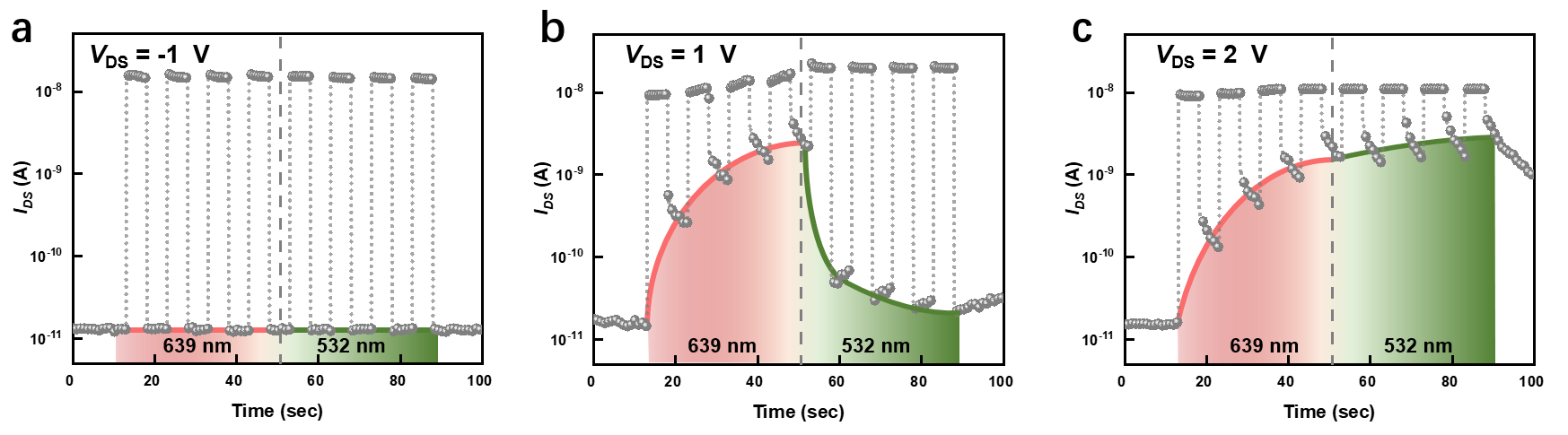


**Fig. S6.** **a-c)** Time-resolved photocurrent following illumination with four pulses each of 639 nm and 532 nm wavelengths (both at a light intensity of 1 mW·cm^2^, 5 second duration, and 50% duty cycle), measured at various applied biases: -1 V (a), 1 V (b), and 2 V (c). This highlights the device’s potential for bidirectional operation at *V*_DS_ = 1 V.


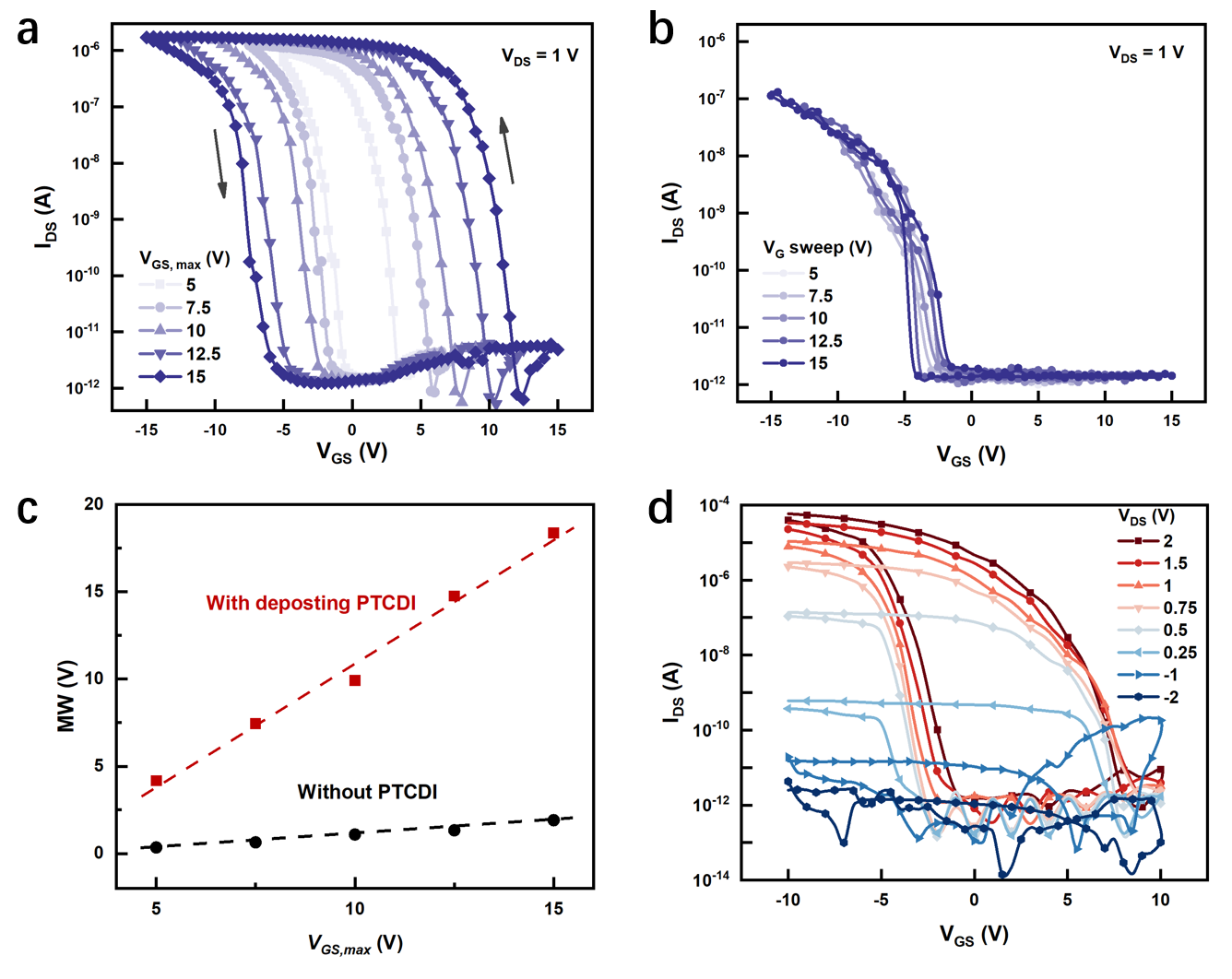


**Fig. S7**. **a-b)** Transfer curves of the Au offset-contacted WSe_2_ transistor: **(a)** after and **(b)** before the deposition of patterned PTCDI-C_13_ doping layers. The curves were obtained under dual sweeping of *V_GS_* ranging from ±5 to ±15 V, with a fixed bias of *V_DS_* = 1 V. **c)** Memory window width as a function of maximum *V_GS_* of the device before (black dots) and after (red dots) the deposition of PTCDI-C_13_. A significantly larger maximum width of ~18.5 V is achieved at a V_GS_ sweep of ±15 V for the PTCDI-doped device, compared to the undoped device (~1.5 V). **d)** Transfer curves of the PTCDI-doped device under a dual sweeping of *V_GS_* of ±10 V, with varying *V_DS_* from -2 V to 2 V.


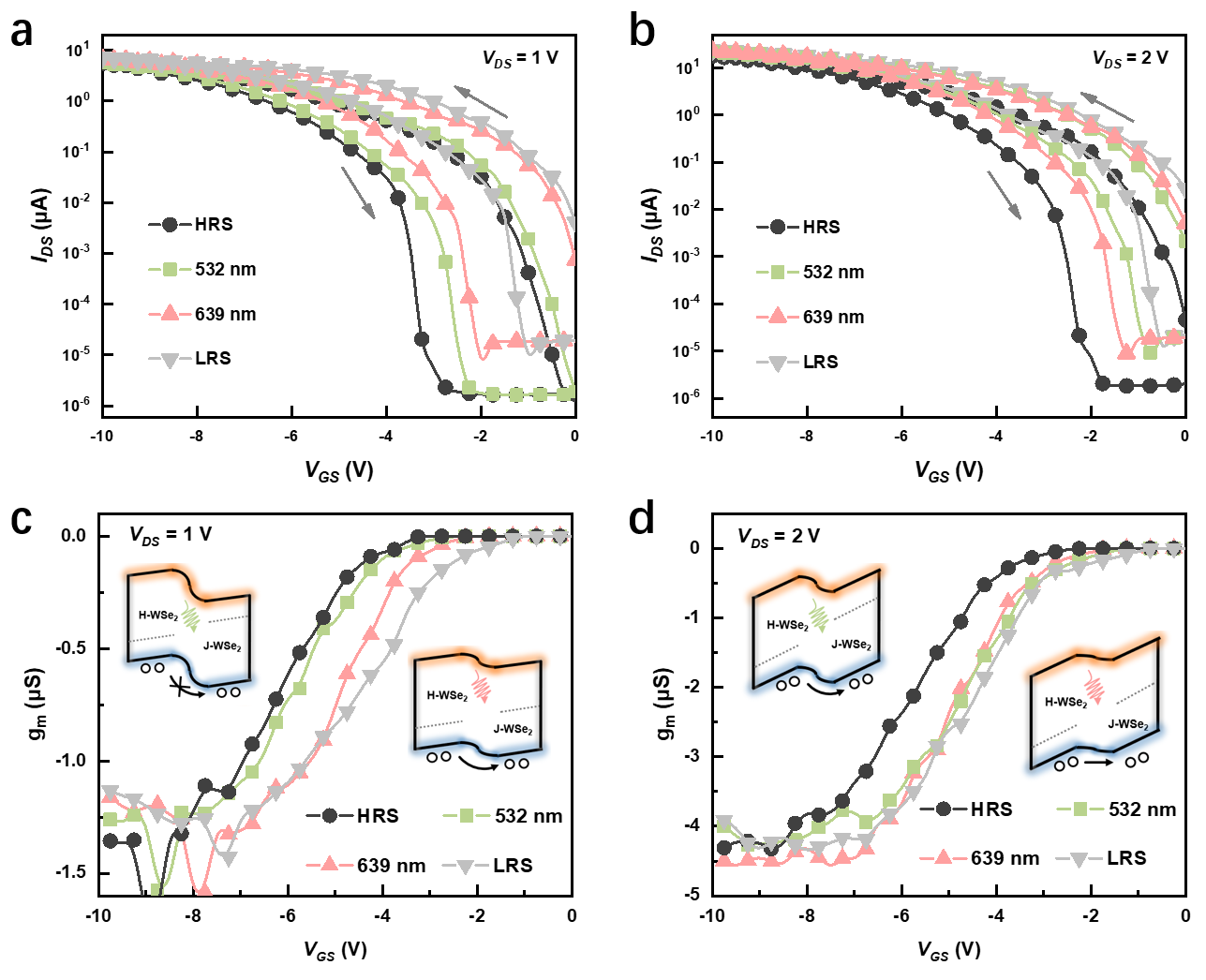


**Fig. S8**. **Transconductance analysis**. **a-b)** Transfer curves following exposure to different light and electric pulses, with *V_GS_* dual swept from 0 V to -10 V and back to 0 V at *V_DS_* of 1 V **(a)** and 2 V **(b)**. **c-d)** Extracted transconductance curves from the *V_GS_* sweep ranging from 0 V to -10V at *V_DS_* of 1 V **(c)** and 2 V **(d)**. The insets illustrate the evolution of the junction barrier (*Φ*_WSe2_) under varying incident wavelengths and bias voltages.


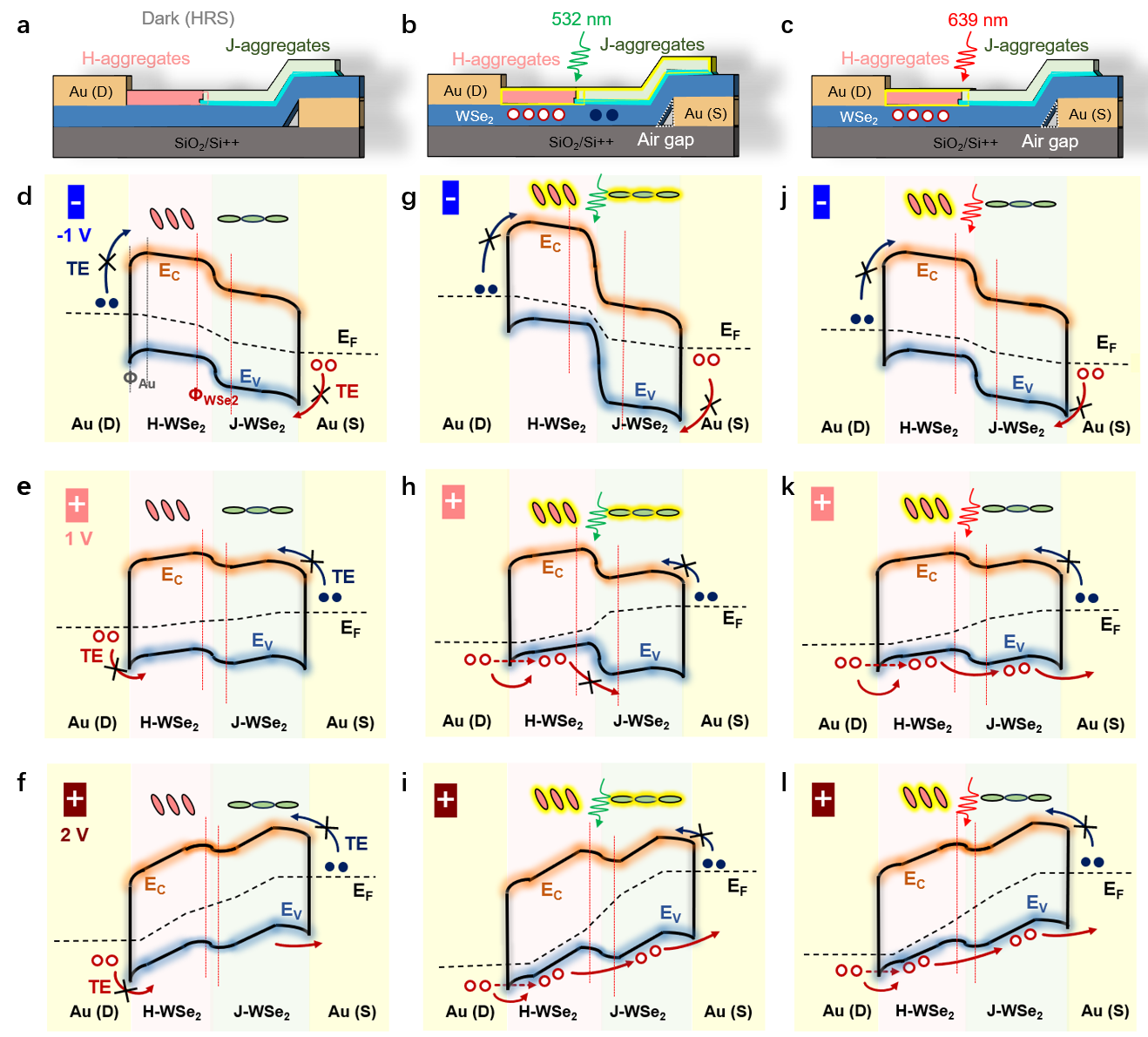


**Fig. S9. a-c)** Photogating effects of J- and H-aggregated PTCDI-C_13_ on the WSe_2_ channel under dark conditions **(a)**, as well as under 532 nm **(b)** and 639 nm **(c)** illumination. **d-l)** Schematic band alignments of the device, illustrating the evolution of the band structure under different wavelengths: dark conditions **(d-f)**, 532 nm illumination **(g-i)** and 639 nm illumination **(j-l)**, and bias voltages: *V*_DS_ = -1 V **(d, g, j)**, *V*_DS_ = 1 V **(e, h, k)** and *V*_DS_ = 2 V **(f, i, l)**.


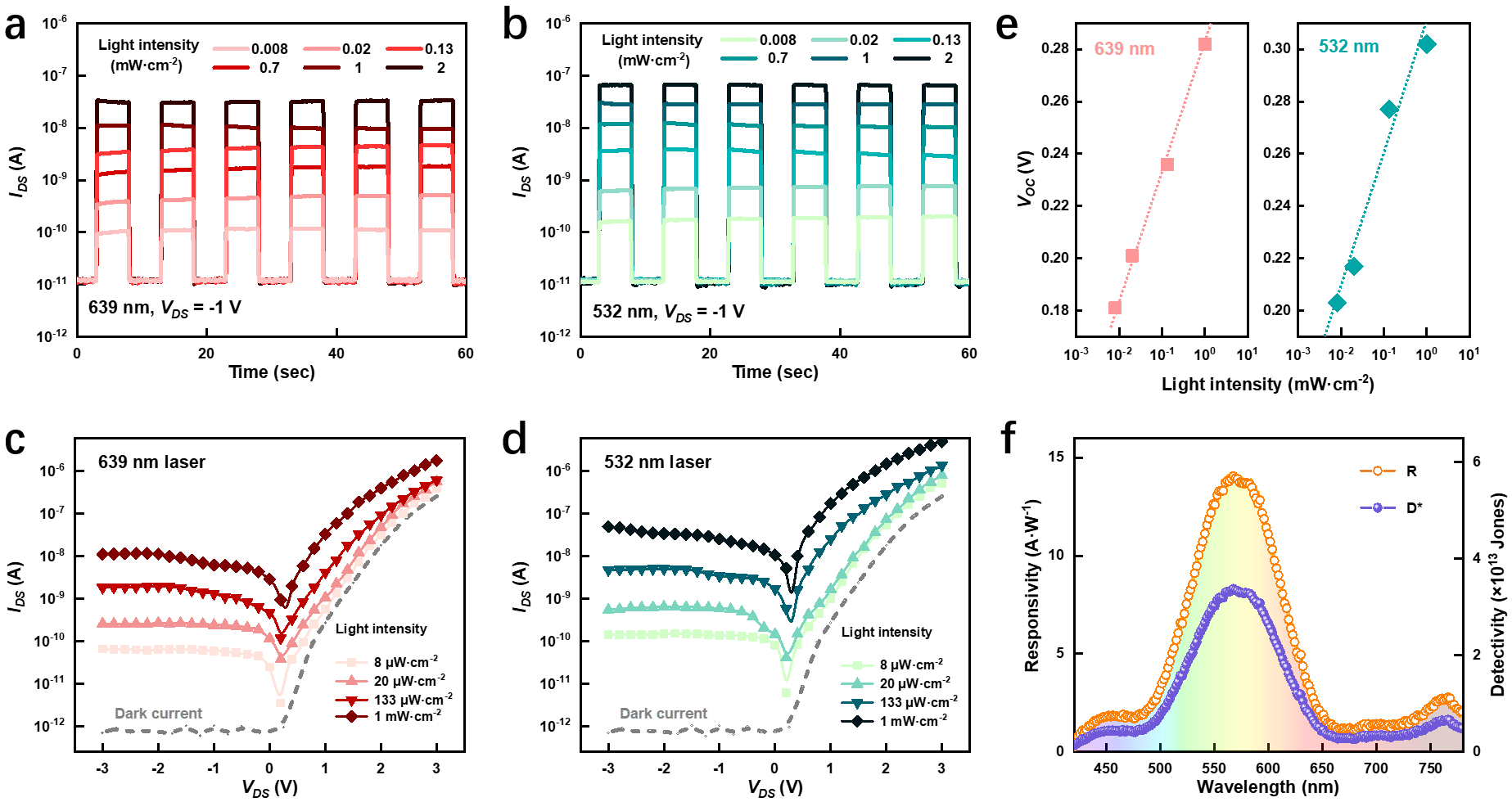


**Fig. S10**. **a-b)** Time-resolved photocurrent curves of the PTCDI-C_13_ doped WSe_2_ diode under laser illumination of 639 nm **(a)** and 532 nm **(b)** with varying light intensity ranging from 0.008 mW·cm^-2^ to 2 mW·cm^-2^. **c-d)** Photocurrent *I_DS_-V_DS_* curves of the device under laser illumination of 639 nm **(c)** and 532 nm **(d)** with varying light intensity. **e)** Open-circuit voltage as a function of light intensity of 639 nm and 532 nm laser illuminations. **f)** Responsivity and detectivity curves measured under light wavelength sweeping from 400 nm to 800 nm at *V_DS_* = -1 V.


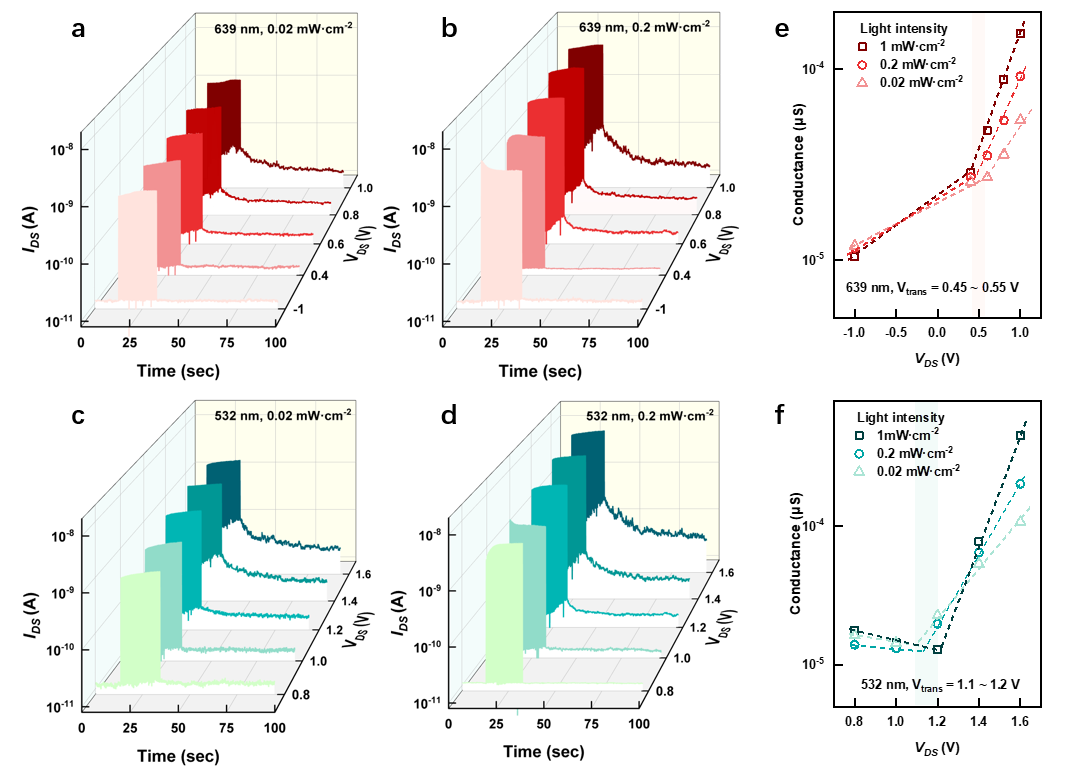


**Fig. S11.** **a-b)** Time-resolved photocurrent curves for 50 pulses (5 Hz, 50% duty cycle) of 639 nm illumination at light intensities of 0.02 mW·cm^2^ **(a)** and 0.2 mW·cm^2^ **(b)** under varying *V_DS_* from -1 V to 1 V, both revealing a volatile to non-volatile transition at *V_DS_* = 0.6 V. **c-d)** Time-resolved photocurrent curves for 50 pulses (5 Hz, 50% duty cycle) of 532 nm illumination at light intensities of 0.02 mW·cm^-2^ **(c)** and 0.2 mW·cm^-2^ **(d)** under varying *V_DS_* from 0.8 V to 1.6 V, both revealing a volatile to non-volatile transition at V_DS_ = 1.2 V. **e-f)** Conductance as a function of *V_DS_* extracted from the postsynaptic current at a fixed decay time of 0.2 sec for 639 nm **(e)** and 532 nm **(f)** illumination, with light intensities varying from 0.02 mW·cm^-2^ to 2 mW·cm^-2^.


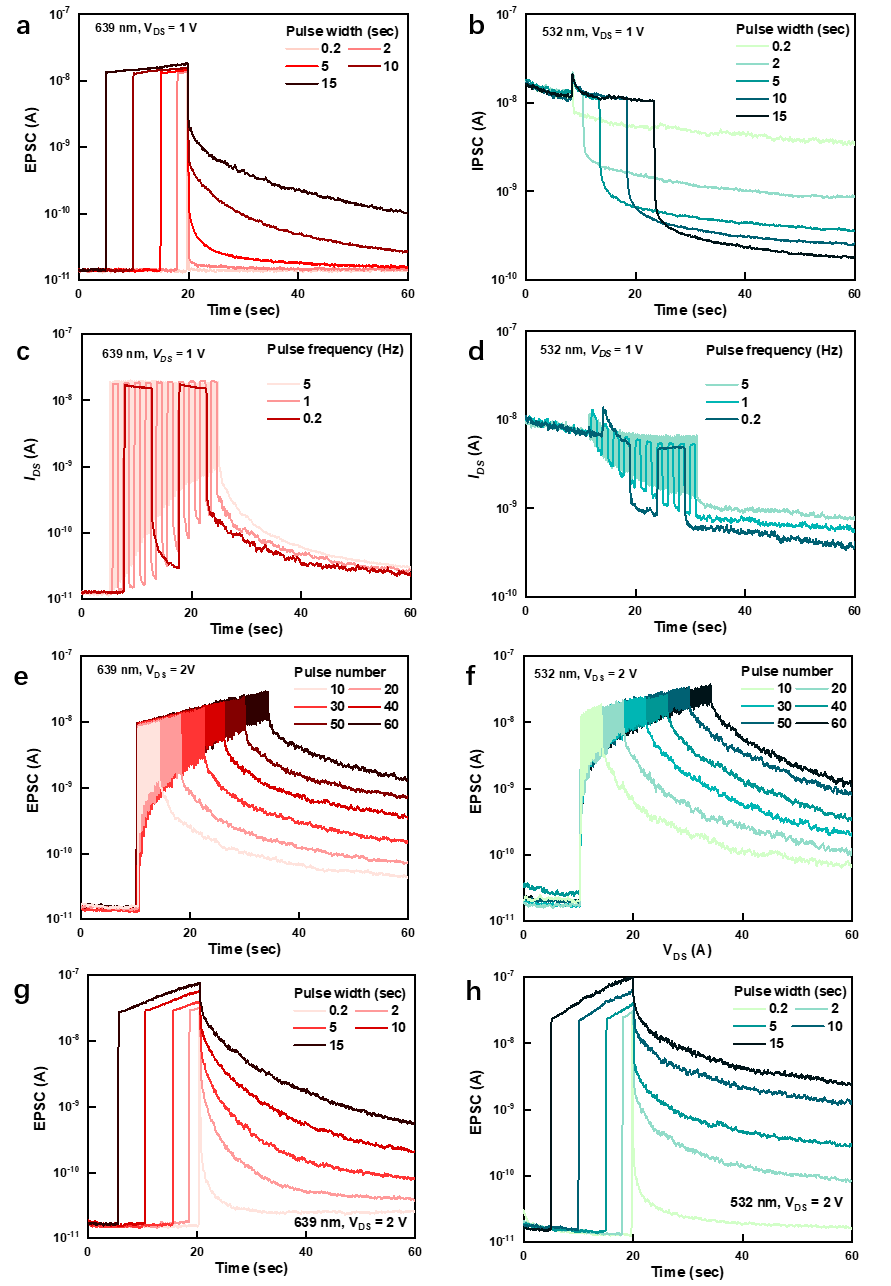


**Fig. S12**. **a-b)** Postsynaptic current triggered by a single light pulse of varying durations from 0.2 s to 15 s at *V_DS_* = 1 V under 639 nm **(a)** and 532 nm **(b)** illuminations. **c-d)** Postsynaptic current triggered by pulses of varying frequencies from 0.2 Hz to 5 Hz at a duration of 20 seconds under 639 nm **(c)** and 532 nm **(d)** illuminations, biased at *V_DS_* = 1 V.

**e-f)** Postsynaptic current triggered by continuous light pulses (5 Hz, 50% duty cycle) of varying numbers from 10 to 60 at *V_DS_* = 2 V under 639 nm **(e)** and 532 nm **(f)** illuminations. **g-h)** Postsynaptic current triggered by a single light pulse of varying durations from 0.2 s to 15 s at *V_DS_* = 2 V under 639 nm **(g)** and 532 nm **(h)** illuminations. The light intensity is fixed at 1 mW·cm^-2^.


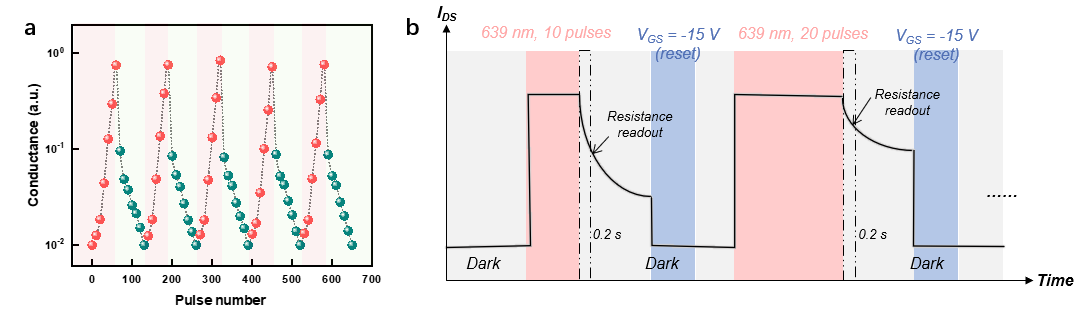


**Fig. S13.** Schematic illustration of the programming and erasing processes, along with the readout points for measuring cyclic resistance states.


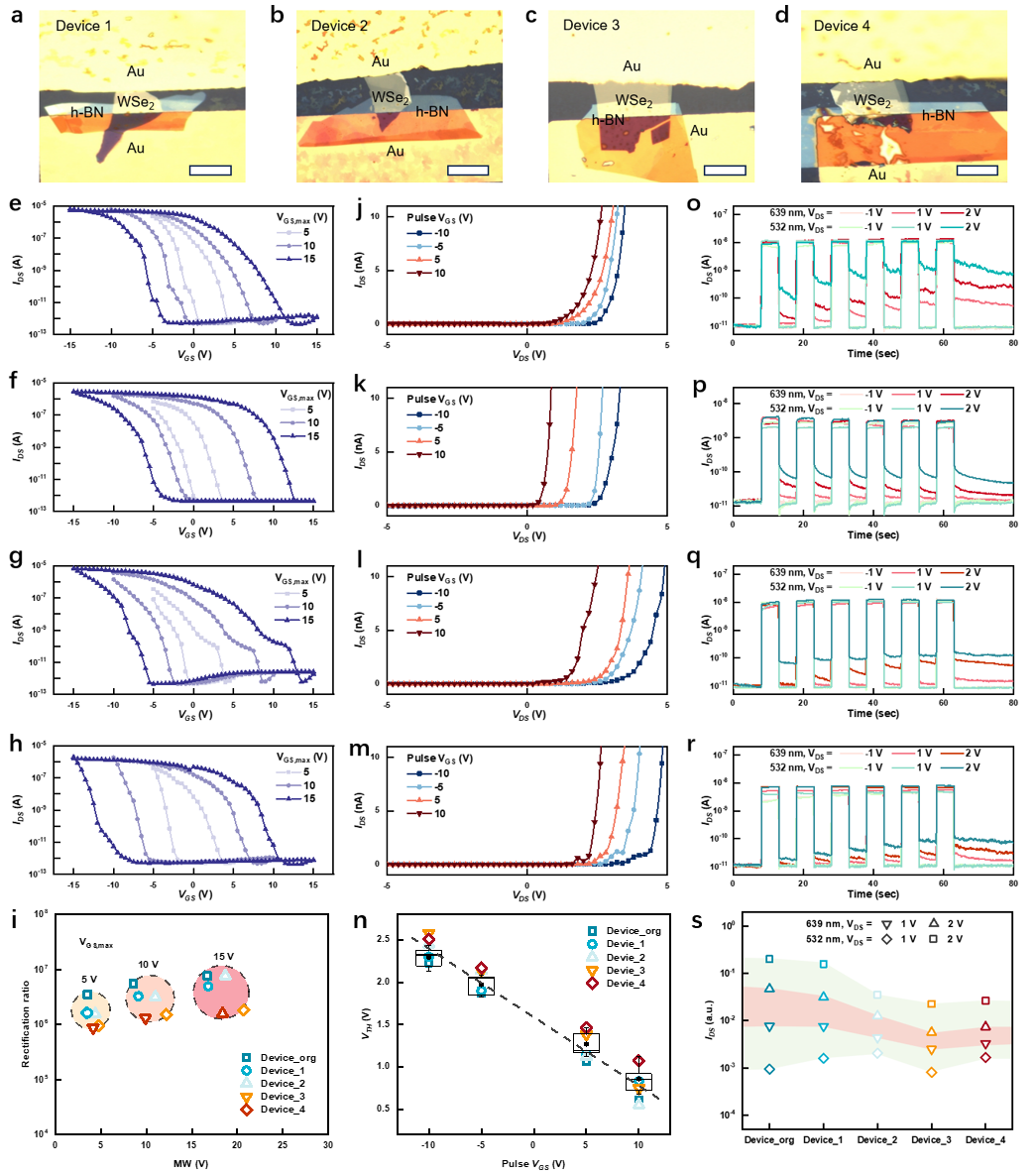


**Fig. S14.** **a-d)** Optical images of PTCDI-C_13_ patterned doped WSe_2_ photodiodes labeled *Device 1* through *Device 4*. Scale bar: 10 μm. **e-h)** Transfer characteristic curves for *Device 1* **(e)**, *Device 2* **(f)**, *Device 3* **(g)**, and *Device 4* **(h)** at varying *V*_GS_ sweeping ranges. **i)** Memory window width and rectification ratio extracted from transfer curves at varying V_GS_ sweeping ranges. **j-m**) Electrically tunable *V*_TH_ shifts observed in the *I*_DS_*-V*_DS_ curves after applying V_GS_ pulses ranging from -10 V to 10 V with a duration of 2 sec for *Device 1* **(j)**, *Device 2* **(k)**, *Device 3* **(l)**, and *Device 4* **(m)**. **n)** Statistics of the extracted *V*_TH_ values as a function of pulsed gate voltages. **o-r**) Time-resolved photocurrent measurements during continuous pulse illuminations (5 s, 50% duty cycle, 1 mW·cm^-2^) at wavelengths of 639 nm and 532 nm, for *Device 1* **(m)**, *Device 2* **(n)**, *Device 3* **(o)**, and *Device 4* **(p)**. **s)** Extracted photocurrent measured at a delay window of 0.2 seconds following pulses from 639 nm to 532 nm at V_DS_ of 1 V and 2 V.


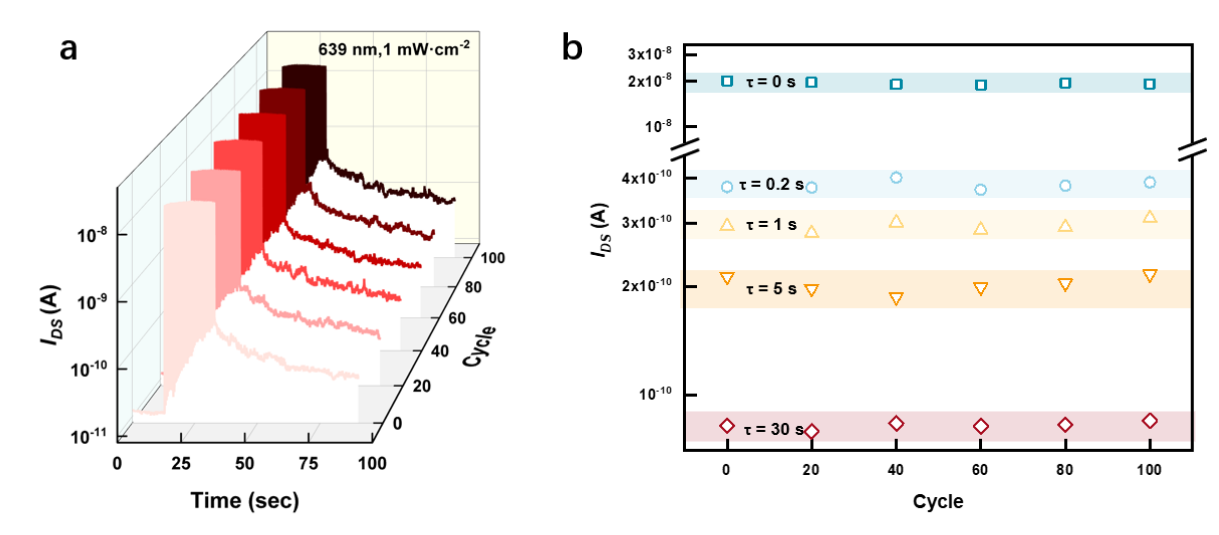


**Fig. S15.** **a)** Time-resolved photocurrent curves under 639 nm illumination at *V_DS_* = 1.4 V for cycle numbers ranging from 0 to 100. Each cycle is programmed with 50 laser pulses (1 mW·cm^2^, 5 Hz, 50% duty cycle) and reset using *V_GS_* pulses at -15 V for 2 seconds. **b)** Extracted postsynaptic current at varying decay times from 0 seconds to 30 seconds for different cycle numbers, indicating robust endurance in reconfigurability.


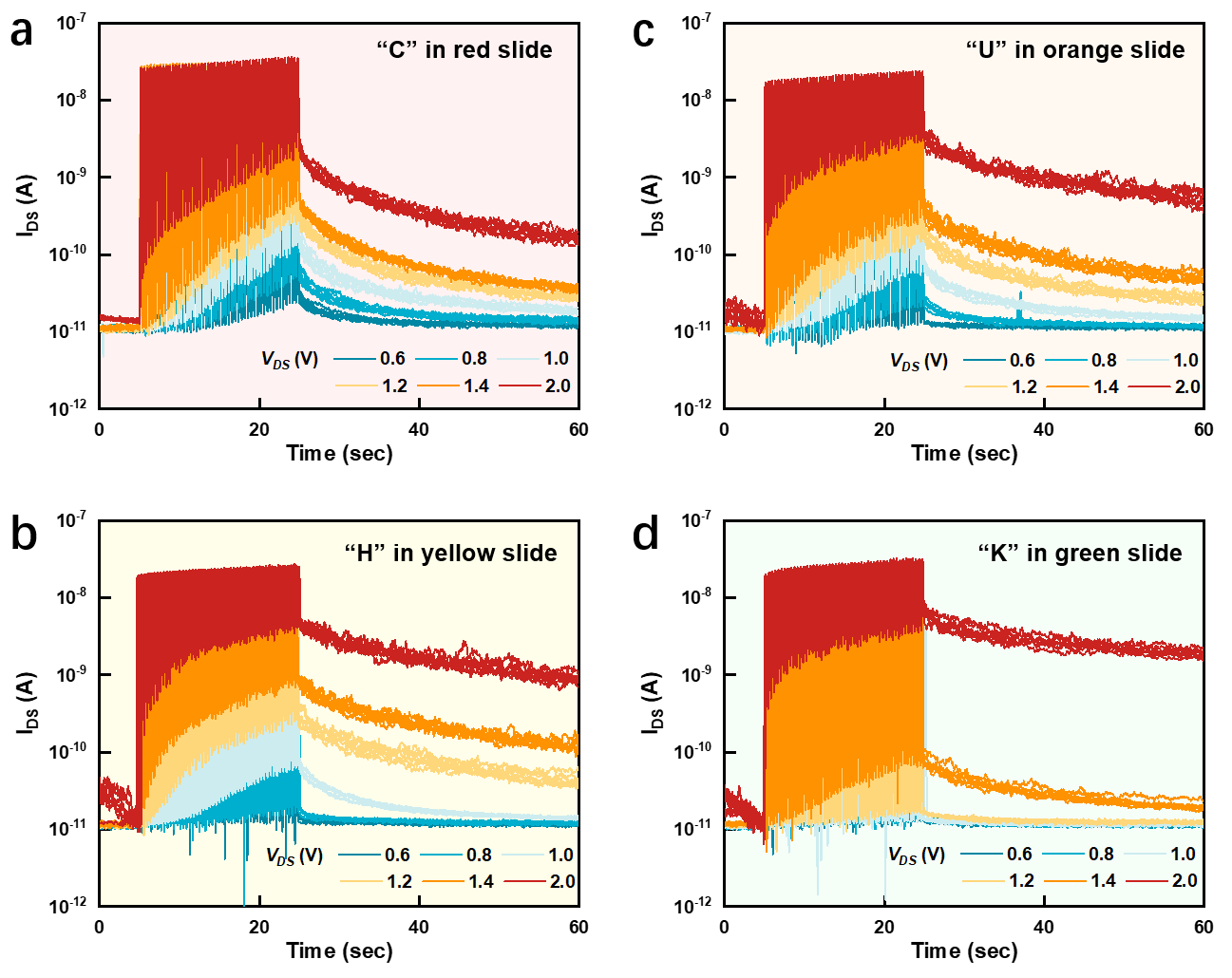


**Fig. S16**. **a-d)** Time-resolved photocurrent curves recorded at different *V_DS_* values in single pixel imaging applications. Each curve corresponds to the device being triggered by 50 white light pulses (1 mW·cm⁻², 5 Hz, 50% duty cycle), filtered through slides patterned with the characters "C" in red **(a)**, "U" in orange **(b)**, "H" in yellow **(c)**, and "K" in green **(d)**, respectively. Erasing pulses (-15V, 2 s) were regularly applied to the bottom gate to initialize the resistance state before recording each pixel.


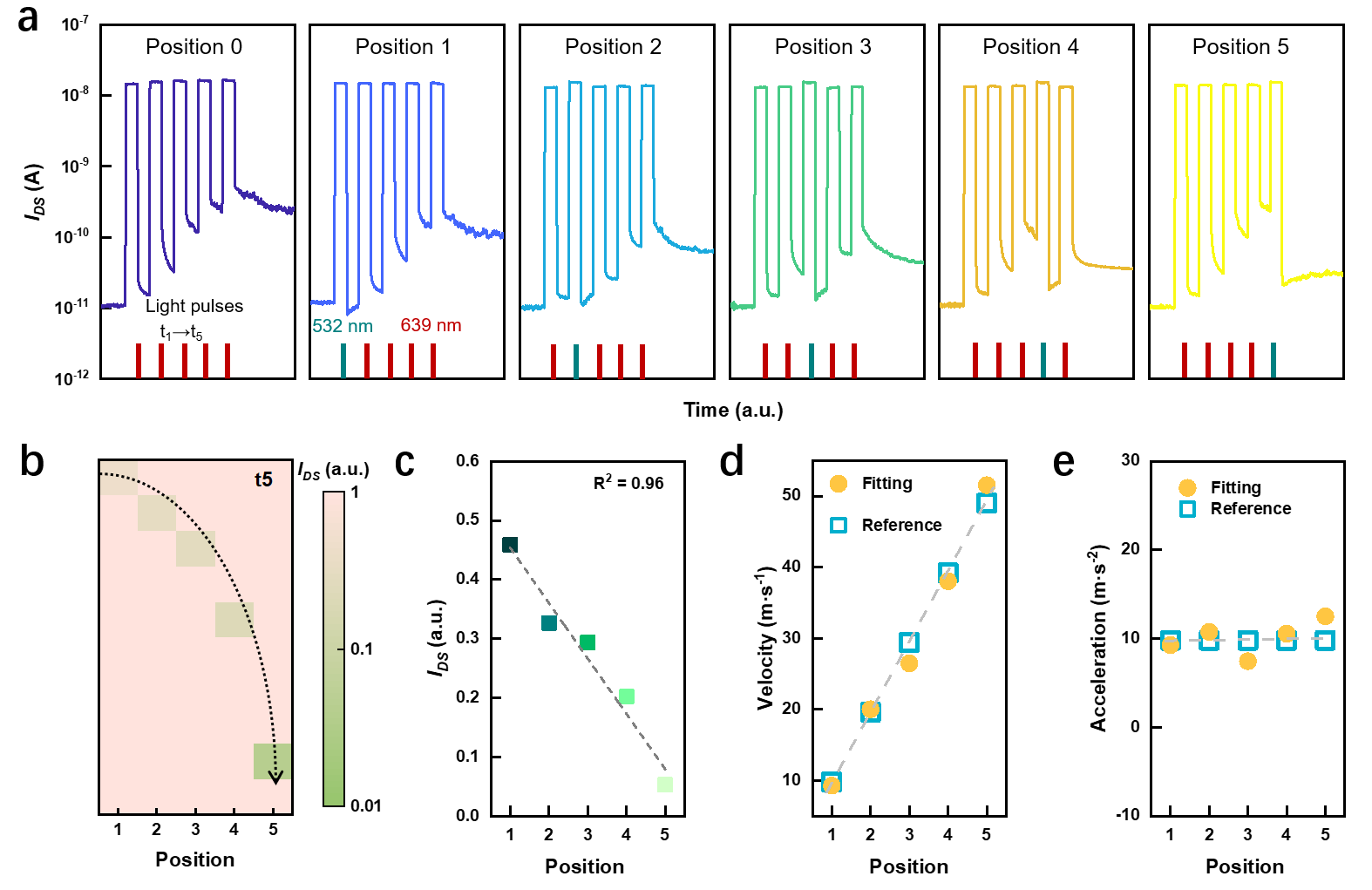


**Fig. S17.** **a)** Postsynaptic current triggered by 532 nm/639 nm mixed light pulses (1 mW·cm^-2^, 5 s, 50% duty cycle) with varying sequencies. **b)** Current mapping measured at t_5_, illustrating the motion trajectory and direction of the object. **c)** Extracted normalized current from **(b),** exhibiting an ideal linear tendency in current decay from *position 1* to *position 5*. **d-e)** Fitted velocity **(d)** and acceleration **(e)** at different potions derived from current data, corresponding to the actual motion parameters.


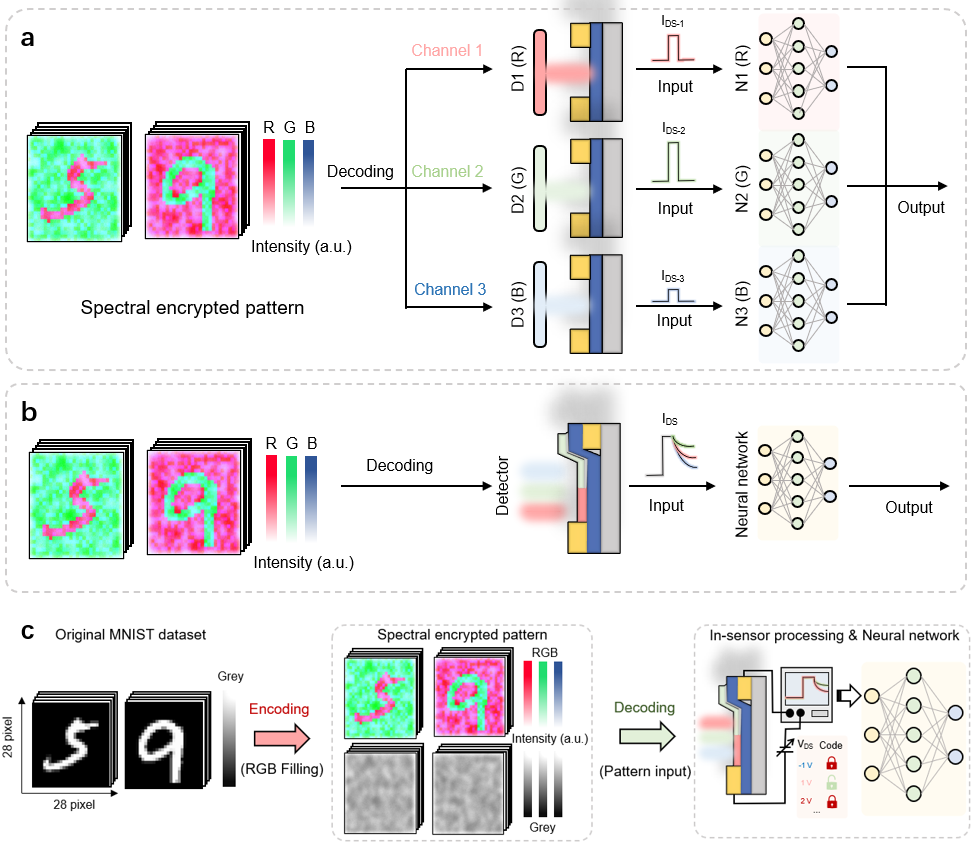


**Fig. S18. a-b)** Schematic illustration of the spectral decoding task using multi-channel photodetector systems **(a)** and the reconfigurable photodiode **(b)**.


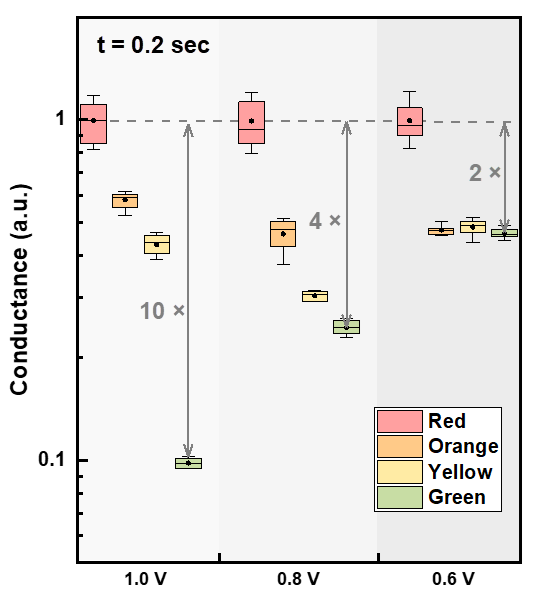


**Fig. S19.** Statistics of the device conductance extracted at a fixed delay window of 0.2 sec for distinct incident colors under varying *V*_DS_ values. The results highlight the device’s potential to boost throughput via bias modulation without compromising color selectivity.


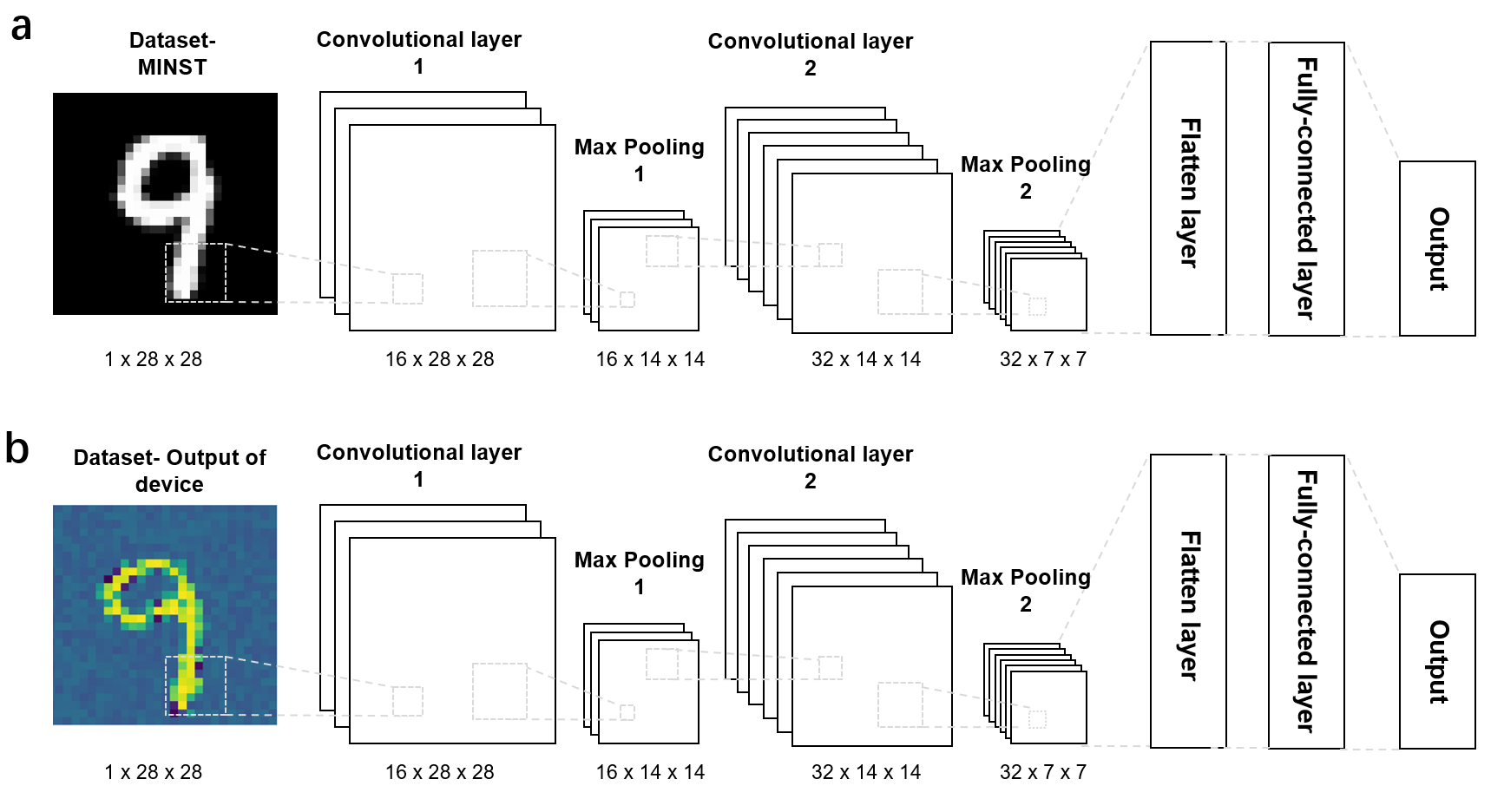


**Fig. S20.** **a-b)** Detailed architectures of the convolutional neural network (CNN) for recognizing the grayscale MNIST dataset **(a)** and for interpreting device output signals into color-coded patterns **(b)**.


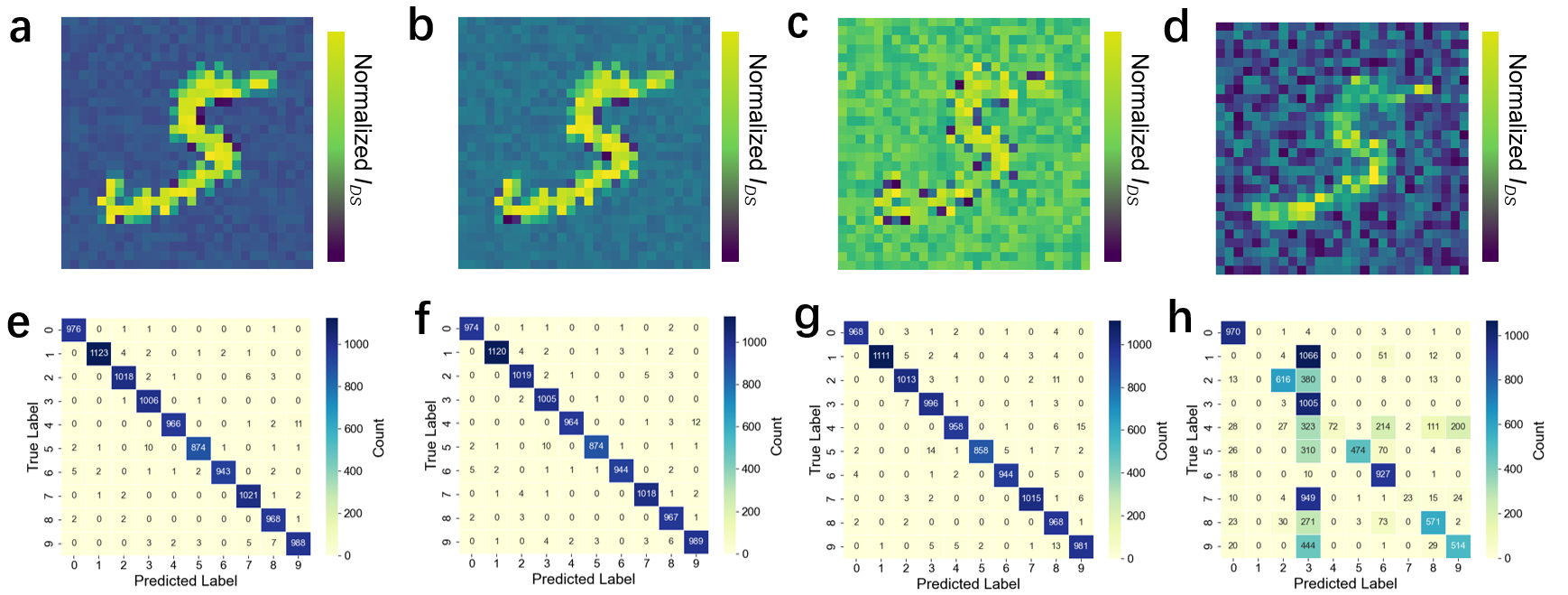


**Fig. S21**. Simulation result of the device in green-filter mode at *V_DS_* = 1 V: **a-d)** Photocurrent mapping obtained at varying decay times of 0.2 s **(a)**, 1 s **(b)**, 5 s **(c)** and 30 s **(d)**. **e-h)** Corresponding confusion matrices showing a decrease in recognition accuracy of 98.99% **(e)**, 98.90% **(f)**, 98.28% **(g)** and 51.80% **(h)** at 0.2 s, 1 s, 5 s and 30 s, respectively.


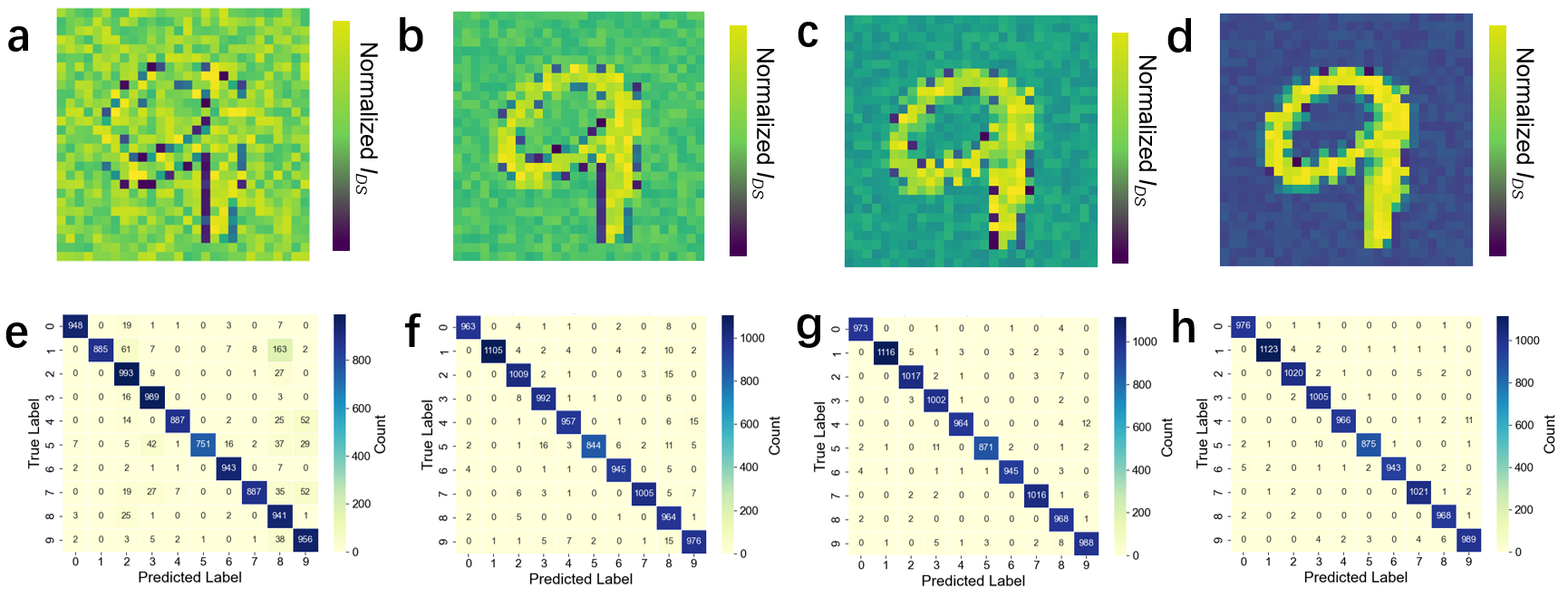


**Fig. S22**. Simulation result of the device in red-filter mode at *V_DS_* = 2 V. **a-d)** Photocurrent mapping obtained at varying decay times of 0.2 s **(a)**, 1 s **(b),** 5 s **(c)** and 30 s **(d)**. **e-h)** Corresponding confusion matrices showing an increase in recognition accuracy of 91.95% **(e)**, 97.76% **(f)**, 98.76% **(g)** and 99.02% **(h)** at 0.2 s, 1 s, 5 s and 30 s, respectively.

| **Device** | **λ (nm)** | **I_on_/I_off_** | **R_max_ (A·W^-1^)** | **D^*^_max_ (Jones)** | **τ_r_ (ms)** | **τ_d_ (ms)** |
| --- | --- | --- | --- | --- | --- | --- |
| PTCDI-doped WSe_2_ (**this work**) | 425-775 | 7.85×10^4^ | 14.1 | 3×10^13^ | ~0.056 | ~0.065 |
| WSe_2_ DGFET ^[1]^ | 442-940 | ~ 50 | 7×10^-4^ | --- | 10.4 | 9.8 |
| Monolayer WSe_2_ ^[2]^ | 650-970 | --- | 0.36 | 1×10^9^ | 310 | 930 |
| Au-WSe_2_-Gr ^[3]^ | 650 | 10^8^ | 7550 | 3×10^12^ | 0.332 | 0.554 |
| WSe_2_/Gr ^[4]^ | 390-1080 | 10-100 | 6.66×10^-3^ | 1.98×10^8^ | 800 | 1400 |
| WSe_2_/MoS_2_ ^[5]^ | 532 | ~ 100 | 0.3 | --- | 5 | 10 |
| WSe_2_/ZnO ^[6]^ | 300-850 | 10^2^~10^3^ | ~ 3 | 1.39×10^11^ | 0.496 | 0.515 |
| WSe_2_/PbS QDs ^[2]^ | 970 | ~ 10 | 2×10^5^ | 7×10^13^ | 7 | 480 |
| PPh3-doped WSe_2_ ^[7]^ | 520 | 10-100 | 10^5^-10^6^ | --- | 2.8 | 20.8 |
| O-doped WSe_2_ ^[8]^ | 365-1064 | 2208 | 0.106 | 1.52×10^12^ | 0.059 | 0.018 |
| F_4_-TCNQ-doped WSe_2_/ReS_2_ ^[9]^ | 785 | ~ 10^4^ | 0.29 | 8.02×10^12^ | 0.008 | 0.006 |
| PEI-doped WSe_2_ ^[10]^ | 500-1000 | 10^6^ | 80 | 10^11^ | 0.2 | 0.016 |

**Tab. S1**. Performance comparisons between our device (grey background) and various WSe_2_-based photodetectors featuring different structures: Schottky contacted WSe_2_ channels (blue background), WSe_2_ p-n heterostructures combined with other semiconductors (green background), and surface-doped WSe_2_ homo- and heterostructures (orange background).

**References:**

1. J. Groenendijk, *et al*. Photovoltaic and photothermoelectric effect in a double-gated WSe_2_ device. *Nano Lett.* 14, 5846-5852 (2014).
2. C. Hu, *et al*. Synergistic effect of hybrid PbS quantum dots/2D‐WSe_2_ toward high performance and broadband phototransistors. *Adv. Funct. Mater.* 27, 1603605 (2017).
3. C. Zhou, *et al*. Self-driven WSe_2_ photodetectors enabled with asymmetrical van der Waals contact interfaces. *npj 2D Mater. Appl.* 4, 46 (2020).
4. M. Pataniya, *et al*. Low cost and flexible photodetector based on WSe_2_ Nanosheets/Graphite heterostructure. *Synth. Met.* 265, 116400 (2020).
5. W. Deng, *et al*. High‐Performance Photodiode Based on Atomically Thin WSe_2_/MoS_2_ Nanoscroll Integration. *Small* 15, 1901544 (2019).
6. H. Ghanbari, *et al*. Multilayer WSe_2_/ZnO heterojunctions for self-powered, broadband, and high-speed photodetectors. *Nanotechnology* 34, 285207 (2023).
7. S. Jo, *et al*. A high‐performance WSe_2_/h‐BN photodetector using a triphenylphosphine (PPh_3_)‐based n‐doping technique. *Adv. Mater.* 28, 4824-4831 (2016).
8. D. Liu, *et al*. WSe_2_ Interdigitated p‐n Homojunction for Broadband High‐Performance Imaging Detection through Localization Effect Synergizes. *Adv. Funct. Mater.* e31930 (2026).
9. Y. Zhan, *et al*. High-performance self-powered WSe_2_/ReS_2_ photodetector enabled via surface charge transfer doping. *ACS Appl. Mater. Inter.* 15, 55043-55054 (2023).
10. Y. Tang, *et al*. WSe_2_ photovoltaic device based on intramolecular p–n junction. *Small* 15, 1805545 (2019).
